# An improved *Sms* p.Gly56Ser model of Snyder–Robinson syndrome reveals phenotypic parallels with clinical features

**DOI:** 10.1242/dmm.052953

**Published:** 2026-09-23

**Authors:** Tracy Murray Stewart, Saurabh Tata, Pierre-Alexandre Piec, Jackson R. Foley, Teri Koerner, Mary Jo Kutler, Anthony E. Pegg, Charles E. Schwartz, Robert A. Casero, Aamir Zuberi, Cathleen Lutz, Maximiliano Presa

**Affiliations:** ^1^ Sidney Kimmel Comprehensive Cancer Center, Johns Hopkins School of Medicine, Baltimore, MD 21287, USA; ^2^ Rare Disease Translational Center, The Jackson Laboratory, Bar Harbor, ME 04609, USA; ^3^ Snyder-Robinson Foundation, McLean, VA 22101, USA; ^4^ Department of Cell and Biological Systems, Pennsylvania State University College of Medicine, Hershey, PA 17033, USA; ^5^ International Center for Polyamine Disorders, Department of Pediatrics and Human Development, College of Human Medicine, Michigan State University, Grand Rapids, MI 49503, USA

**Keywords:** Snyder–Robinson syndrome, Polyaminopathy, Polyamine, Spermidine, Spermine, Rare disease

## Abstract

Snyder–Robinson syndrome (SRS) is a rare X-linked disorder, caused by pathogenic variants in spermine synthase (SMS), associated with spermine deficiency and excessive spermidine accumulation. Previously reported mouse models exhibited reduced birthrate and survival of affected males, greatly limiting their experimental utility. Here, we describe a new mouse model, carrying the clinically relevant *Sms* p.Gly56Ser (*Sms^G56S^*) allele, in which viable males are recovered at Mendelian ratios, enabling generation of adequately powered cohorts. Hemizygous males produced markedly reduced SMS protein across tissues, recreating the biochemical hallmark of SRS, an elevated spermidine:spermine ratio. *Sms^G56S/Y^* males exhibited reduced body size, altered body composition, decreased locomotor and exploratory behaviors, and reduced seizure threshold, aligning with clinical features reported in patients with SRS. Serum low-density lipoprotein, high-density lipoprotein and cholesterol levels were reduced, while brain histology revealed modest region-specific astrocytic changes. Comprehensive polyamine profiling revealed tissue-specific biochemical disturbances, highlighting putrescine elevation in the brain and informing the development of translational strategies and windows for intervention. Overall, this improved model reproduces multiple key aspects of the human SRS phenotype and provides a robust platform for mechanistic studies and preclinical evaluation of therapies.

## Teaser

**Summary:** An improved mouse model of Snyder–Robinson syndrome is presented, with viable affected males, reduced spermine synthase protein, elevated spermidine:spermine ratios, growth and behavioral deficits, altered body composition and seizure susceptibility, providing a robust platform for therapy testing.

## INTRODUCTION

Snyder–Robinson syndrome [SRS; Online Mendelian Inheritance in Man (OMIM) #309583] results from an inherited deficiency in the spermine synthase (SMS) enzyme. SRS was originally reported in 1969 (Snyder and Robinson, 1969), and the number of confirmed patients with the disorder has substantially increased in recent years owing to increased awareness and access to genomic testing (Civit et al., 2025; Leung et al., 2024; Dontaine et al., 2021; Qazi et al., 2020; Larcher et al., 2020; Albert et al., 2015; Zhang et al., 2013; Peron et al., 2013; Schwartz et al., 2011, 2013; Becerra-Solano et al., 2009; de Alencastro et al., 2008; Cason et al., 2003; Arena et al., 1996). However, SRS remains an ultra-rare disease, and the resources available for its study are limited.

SRS is an X-linked intellectual disability/developmental disorder, primarily affecting males (Schwartz et al., 2013). The enzyme encoded by the *SMS* gene is the sole source of endogenous spermine (SPM) production by mammalian cells. Gene variants affecting the catalytic activity of SMS, therefore, cause deficiency in SPM, while its precursor, spermidine (SPD), accumulates to excessive levels (Fig. 1A). This causes the elevated SPD:SPM ratio that is a biochemical hallmark of SRS (Cason et al., 2003; Murray-Stewart et al., 2018).

The phenotype of SRS is variable, and, although in many cases, phenotype severity correlates with the degree to which the SPD:SPM ratio is perturbed by a specific mutation, this is not always the case. Although numerous *SMS* variants have been documented in individuals with SRS, certain ones have occurred in multiple, unrelated families. The most common of these is a glycine-to-serine change at residue 56 of the SMS protein (SMS^G56S^). This variant is reported to possess minimal SMS enzymatic activity, manifesting as a severe phenotype that includes global developmental delay, profound intellectual disability, persistent hypotonia, seizures and kyphoscoliosis (de Alencastro et al., 2008). As most SRS-associated variants reported to date are hypomorphic rather than complete loss-of-function variants (Larcher et al., 2020; Stewart et al., 2023), an appropriate animal model for the study of SRS is lacking. Current resources for the study of SRS comprise patient-derived lymphoblastoid and fibroblast lines, which, although valuable, may not necessarily reflect the tissues most relevant to the SRS phenotype. A *Drosophila melanogaster* model has also provided important insights into SRS physiology (Tao and Zhai, 2025; Li et al., 2017). The original mouse model of SRS lacked the *Sms* gene entirely, in addition to the neighboring phosphate-regulating endopeptidase X-linked (*Phex*) gene, complicating interpretation of the SMS-dependent phenotype (Wang and Pegg, 2011; Wang et al., 2004). The severe phenotype of this animal, including a propensity for sudden death, provided additional experimental challenges. We, therefore, used gene editing to introduce the *Sms^G56S^* variant into mice, creating the first disease-relevant mouse model of SRS (JAX strain #031170). This model was recently characterized (Akinyele et al., 2024) but was also plagued with limitations, in particular low birthrate of affected males, which hampered its utility for the evaluation of treatment approaches for SRS, of which there are currently none. The studies that follow address the limitations of this model in terms of breeding and survival by transferring the pathogenic *Sms^G56S^* allele onto a hybrid background, vastly improving birthrate and vigor, and providing a reliable model system for the study of SRS. We present a comprehensive characterization of the biochemical, behavioral, physiological and histopathological phenotypes of this new mouse strain, in parallel with those of its predecessor, when possible, and relate it to the clinical characteristics of patients with the same variant, thereby validating it as an appropriate disease model with which to explore the underlying pathophysiology of SRS. These studies additionally reveal important parameters for use in determining potential treatment response and phenotype reversal.

## RESULTS

### Generation, breeding and survival of *Sms^G56S/Y^* mice

Mouse and human SMS proteins share 96% amino acid identity (Fig. S1A), including the glycine residue altered in the pathogenic *SMS^G56S^* clinical variant of SRS. We therefore engineered this missense glycine-to-serine mutation at amino acid 56 in exon 2 of the mouse *Sms* gene, using a CRISPR-Cas9 endonuclease-mediated gene-editing strategy in C57BL/6J zygotes. This *Sms-*targeted editing resulted in two nucleotide changes (underlined) that altered the codon from GGC to TCC (Fig. 1B,C). Germline transmission in C57BL/6J inbred mice resulted in viable heterozygous females capable of producing the *Sms^G56S^* mutant mice when mated with wild-type males, thereby creating the B6-*Sms^G56S^* mouse strain (JAX strain #031170). Western blot analyses revealed dramatically reduced expression of SMS protein in SRS-related tissues of hemizygous male mice, including brain, testicles and quadriceps (skeletal muscle; Fig. 1D; Fig. S1B), consistent with previous reports in brain, triceps and gastrocnemius tissue (Akinyele et al., 2024). Although SMS protein was greatly reduced, it was detected in all tissues examined, suggesting that a low level of enzymatic activity may be maintained. Analysis of heterozygous female carriers (B6-*Sms^+/G56S^*) revealed tissue-specific decreases in SMS protein expression (Fig. S1C). The magnitude of the decrease in brain tissue was far less than that in affected males, although it was statistically significant, while no significant change was detected in skeletal muscle (Fig. S1D). Expression in the ovaries was reduced to less than half that in wild-type females, with no obvious effect on reproductive ability.

**Fig. 1.**
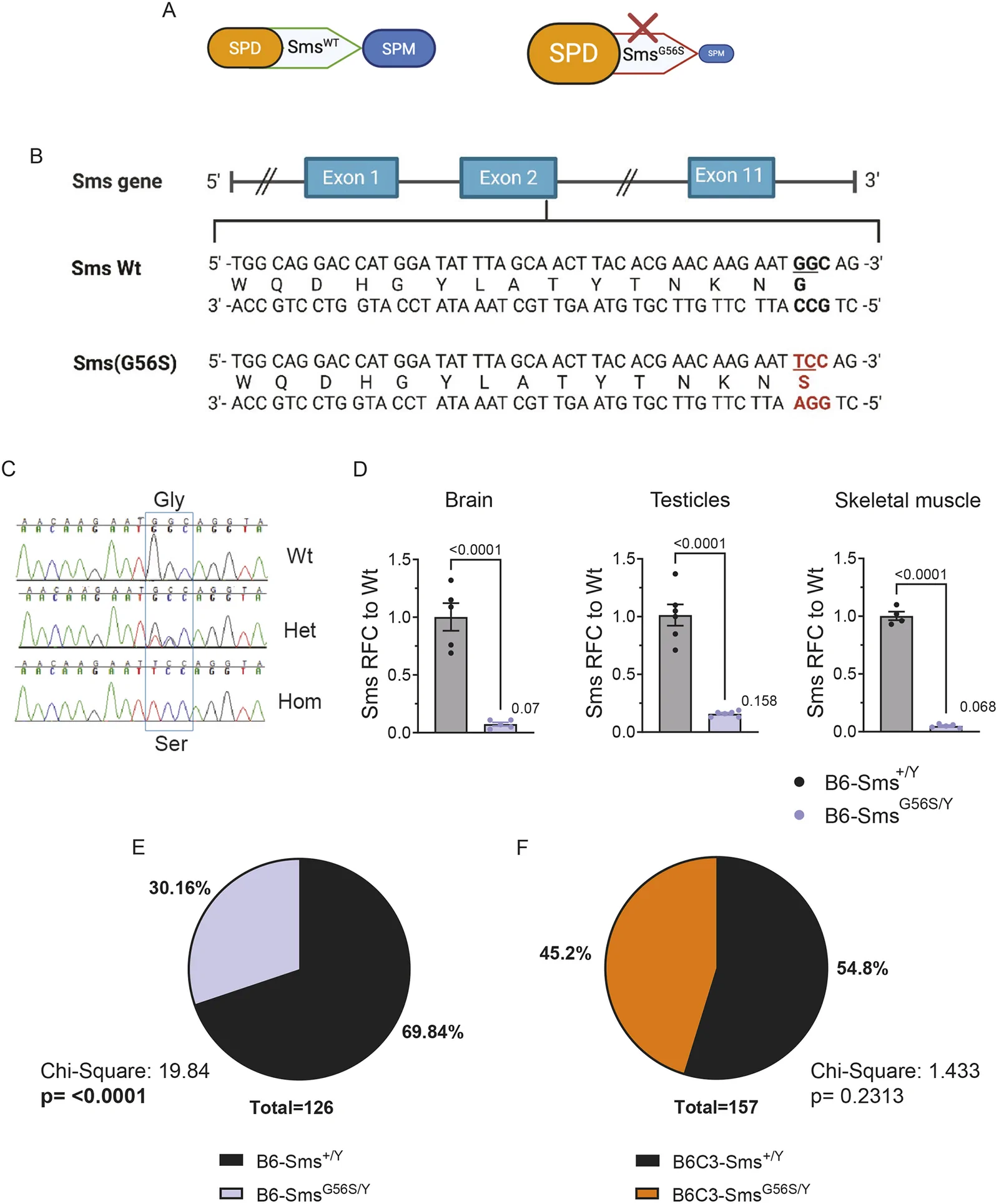
**Development of *Sms^G56S^* mouse strains.** (A) Simplified schematic of the spermine synthase (SMS)-mediated conversion of spermidine (SPD) to spermine (SPM). WT, wild type. Created in BioRender by Stewart, T. M. (2026). https://BioRender.com/va8x4e3. This figure was sublicensed under CC-BY 4.0 terms. (B) CRISPR-Cas9-targeted region in exon 2 producing the *Sms^G56S^* variant allele. (C) Sanger sequencing of the targeted *Sms* region on chromosome X, validating wild-type, heterozygous and targeted alleles. (D) Quantitative representation of SMS protein expression in brain, testicles and skeletal muscle (quadriceps) of 8-week-old B6-Sms^+/Y^ and B6-Sms^G56S/Y^ mice, as determined by western blotting. Data are presented as the means±s.e.m.; *n*=5-6, *P*<0.0001 for brain and gonads and *P*<0.0005 for skeletal muscle (quadriceps). *P*-values were calculated using unpaired two-tailed *t*-test. (E) Representation of the observed survival of male offspring on the pure B6 background; *n*=126, Chi-square 19.84, *P*<0.0001. (F) Representation of the observed survival of male offspring on the hybrid B6C3 background; *n*=157, Chi-square 1.433, *P*=0.2313.

Although the *Sms* variant p.Gly56Ser was successfully generated, the observed number of surviving hemizygous male offspring (*Sms^G56S/Y^*) on the B6 background was significantly less than expected owing to reduced birth rate in addition to high mortality prior to weaning (Fig. 1E and Table 1). With the goal of improving viability, female heterozygous (B6-*Sms^+/G56S^*) mice were crossed with C3H/HeJ male mice (JAX strain #000659), establishing the *Sms^G56S^* variant allele on the ‘B6C3’ hybrid background (JAX strain #033707). Heterozygous females of this new ‘B6C3F1.*Sms^G56S^*’ strain successfully produced viable, hemizygous male pups carrying the variant *Sms^G56S^* allele at nearly the anticipated Mendelian ratio (Fig. 1F). This improvement in the birthrate of hemizygous males, in addition to an 84% survival rate at postnatal day (P)90 compared to 35% in the original strain (Table 1), greatly facilitated the procurement of experimental cohorts of sufficient size for analysis.

**Table 1.** Postnatal survival of male offspring on the C57Bl6/J background

Day	Percentage survival	
	B6-Sms^+/Y^	B6-Sms^G56S/Y^
P0	100	100
P1	100	89
P2	90	68
P3	84	35
P28	84	35
P90	84	35

P, postnatal day.

We subsequently investigated the effects of the *Sms^G56S^* variant allele in both the pure B6 and hybrid B6C3 backgrounds to validate the relevance of each to the other as well as to the human condition. As a recent publication from Akinyele and colleagues elegantly describes several aspects of the phenotype on the B6 background (Akinyele et al., 2024), the studies presented here focus on the B6C3 strain, with reference to parallel studies and complementary data in the original B6 background, when available for comparison.

#### Body weight and composition

The most apparent manifestation of the *Sms^G56S^* allele is its effect on body size. In both the pure and hybrid backgrounds, hemizygous males were significantly smaller than their wild-type male littermates throughout development (Fig. 2A-C; two-way ANOVA with Šidák correction, *P*<0.0001). Although this difference was ∼20-25% at 6 weeks of age, the weight gain of wild-type animals outpaced that of the affected males, resulting in an average deficiency in body weight of 42.7% in the pure B6 strain and 32.4% in the B6C3 hybrid strain at 32 weeks of age (Fig. 2D). Comparing body weights of the two *Sms^G56S^* strains as percentages of those of their wild-type male littermates over time, mice on the pure B6 background overall demonstrated more severe growth deficiency than those on the hybrid background (two-way ANOVA with Šidák correction, *P*=0.0057).

**Fig. 2.**
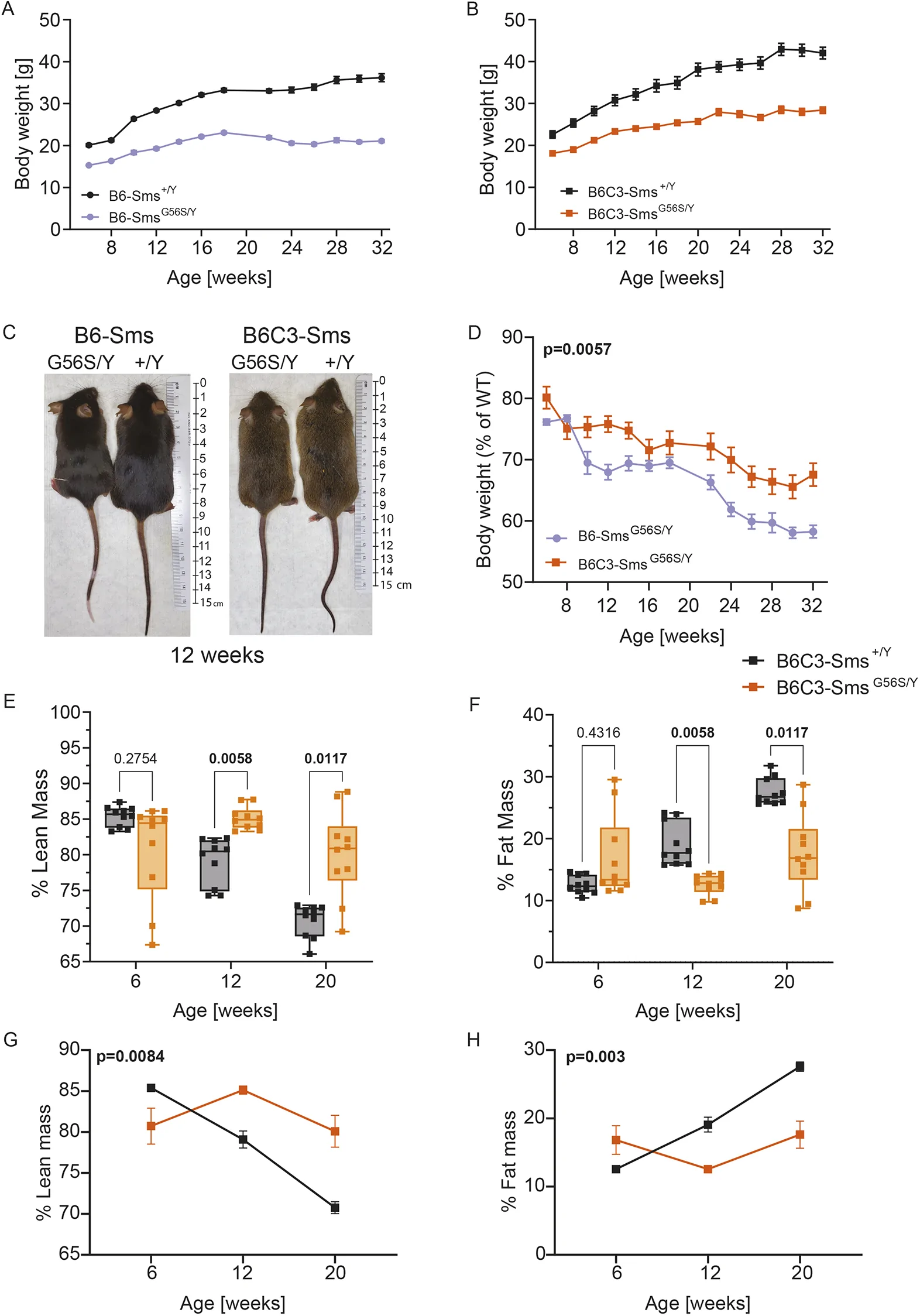
**Alterations in body weight and composition in the Sms^G56S/Y^ mouse models.** (A,B) Total body weight over time of male Sms^G56S/Y^ mice on pure B6 (A) and hybrid B6C3 (B) backgrounds compared to that of wild-type littermates (Sms^+/Y^) of each strain. Data points indicate the average of ten mice/genotype at each time point; error bars indicate s.e.m. (C) Representative 12-week-old male mice showing the notable size difference between littermates with wild-type or G56S *Sms* alleles on both backgrounds. Data points represent the average of ten mice/genotype at each time point; error bars indicate s.e.m. (D) Comparison of the reductions in body weights of the two Sms^G56S/Y^ mouse strains relative to those of their wild-type littermates over time. Significance of the difference between strains was determined by two-way ANOVA with Šidák correction. (E,F) Lean mass (E) and fat mass (F) were measured using dual-energy X-ray absorptiometry (DEXA) in the mixed-background B6C3-Sms^+/Y^ and B6C3-Sms^G56S/Y^ mice at 6, 12 and 20 weeks. Data are presented as percentage of total body weight to adjust for overall size difference; *n*=10 mice/genotype. Data are represented as interleaved box-and-whiskers plots, with boxes representing the 25-75th percentiles and lines indicating the median in individual plots. Whiskers show the minimum and maximum. Adjusted *P*-values were calculated using Wilcoxon matched-pairs signed rank test and Sidak's multiple-comparison method. (G,H) Longitudinal data representing the average percentage of lean mass (G) and fat mass (H) for each genotype over time, with statistics indicating differences between the curves. Two-way ANOVA with Šidák correction revealed significant genotype-driven differences in fat and lean mass as percentages of total tissue mass. Squares represent the average of ten mice/genotype at each time point; error bars indicate s.e.m.

Body composition was analyzed in male mice on both pure and hybrid backgrounds by dual-energy X-ray absorptiometry (DEXA) scan at 20 weeks of age. Additionally, longitudinal scans were obtained for B6C3-Sms^G56S/Y^ mice and their wild-type littermates at 6, 12 and 20 weeks of age. Consistent with the observed reduction in body weight, *Sms^G56S^* mice on both backgrounds had reduced lean mass, fat mass, bone area and bone mineral content measured at 20 weeks of age (Fig. S2A-D). However, when considered relative to total body weight, the percentage of lean mass was significantly increased, whereas fat mass percentage was reduced (Fig. 2E,F; Fig. S2E,F), in SRS mice on both backgrounds, as previously observed in the pure B6 strain (Akinyele et al., 2024). These differences in body composition were absent in mice analyzed at 6 weeks of age. However, the normal age-dependent increase in fat mass and coincidental decrease in lean mass observed in wild-type males (B6C3-Sms^+/Y^) were absent in SRS-affected mice, resulting in a relatively stable body composition throughout development (Fig. 2G,H). DEXA analysis was unable to detect significant differences in bone mineral density (BMD) between the genotypes (Fig. S2G,H), although reduced BMD has previously been reported in B6-Sms^G56S/Y^ animals using more sensitive methodologies (Akinyele et al., 2024).

#### Polyamine profiles in *Sms^G56S^* mice

Biochemically, loss of SMS enzymatic activity leads to a deficiency in intracellular SPM levels with consequential accumulation of its precursor, SPD. The resulting elevation in SPD/SPM ratio has become a defining hallmark of SRS (Fig. 1A; Murray-Stewart et al., 2018). We examined the levels of these polyamines in tissues collected at 11-12 weeks of age and 25-30 weeks of age in both the B6 and B6C3-Sms mice (Fig. 3A,B,D,E,G,H,J,K; Fig. S3A,B). Brain and quadriceps were chosen owing to their association with common manifestations of the syndrome (intellectual disability, seizures and hypotonia); spinal cord was examined as a relatively unaffected component of the nervous system, providing information enriched for neurons for comparison with more heterogenous brain tissue. The SPD/SPM ratio was elevated in all tissues examined (Fig. 3C,F,I,L; Fig. S3C), regardless of age, background strain or involvement in SRS pathology. Although SPM was significantly reduced in all tissues, the accumulation of SPD was generally in excess, leading to an overall increase in total polyamines measured. The exception was in the spinal cord, where, in both B6 and B6C3 strains, significant elevations in SPD were detected in younger mice (11/12 weeks) that were absent in the older cohorts (25/30 weeks, respectively) (Fig. 3D,E; Fig. S3A,B). These data, combined with the extremely low expression levels of SMS protein (Fig. 1D; Fig. S1B), indicate a loss of SMS enzymatic activity throughout tissues of the B6- and B6C3-Sms mouse strains.

**Fig. 3.**
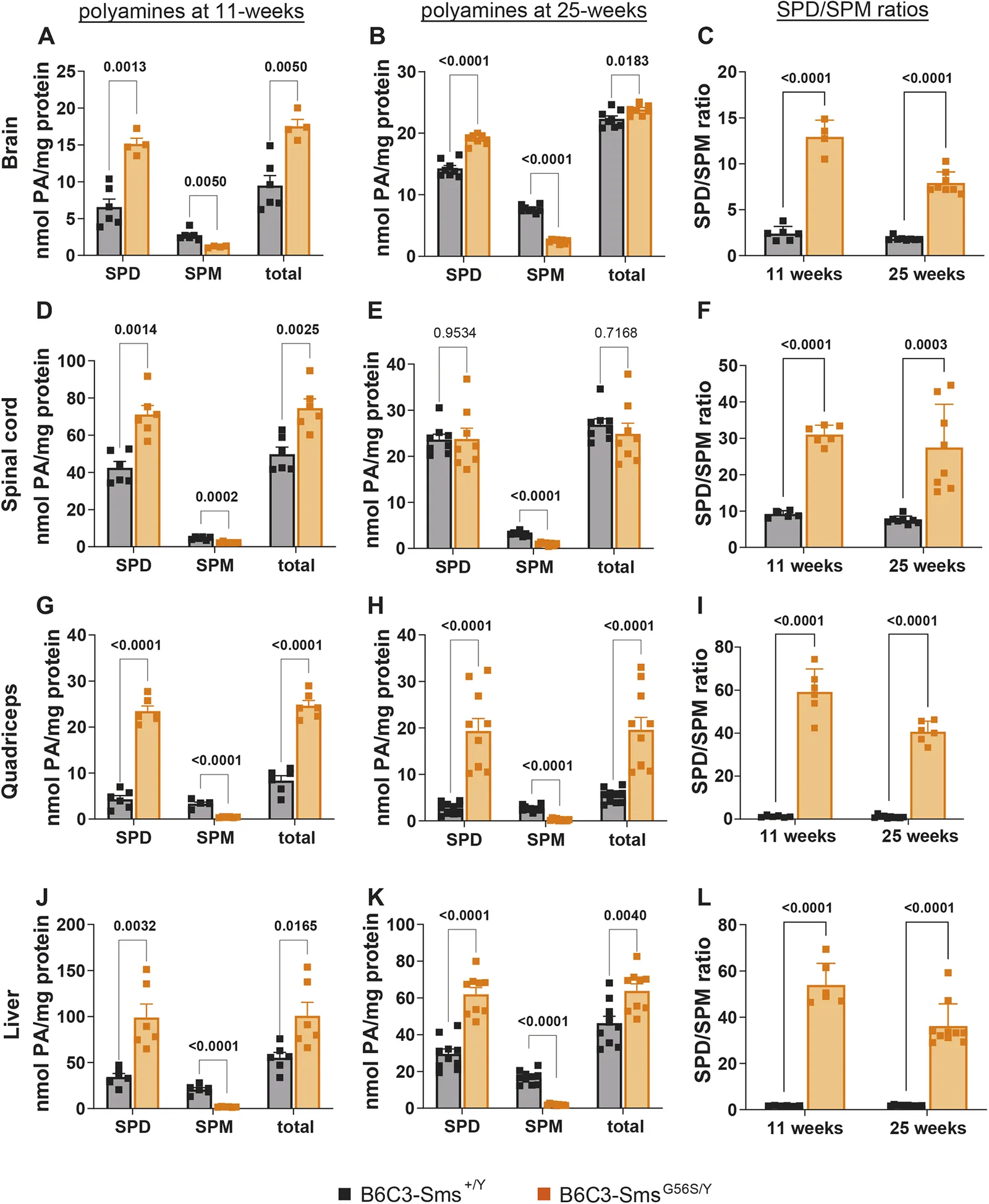
**Dysregulation of polyamine homeostasis in Sms^G56S^ mouse tissues.** (A-L) Concentrations of SPD, SPM, total polyamines and the calculated SPD/SPM ratios were determined in wild-type and SRS (B6C3-Sms^+/Y^ and B6C3-Sms^G56S/Y^, respectively) mouse tissues, including brain (A-C), spinal cord (D-F), quadriceps (G-I) and liver (J-L). Tissues were collected at 11 (*n*=4-6; A,D,G,J) and 25 (*n*=6-10; B,E,H,K) weeks of age. Values on the ordinant are presented as nmol polyamine (PA) measured with normalization to total cellular protein. Resulting SPD/SPM ratios were calculated for each age group (C,F,I,L). Sample sizes for B6C3-SmsG56S/Y mice at 11 weeks of age were as follows: brain, *n*=4; spinal cord, quadriceps and liver, *n*=6. The corresponding sample size for B6C3-Sms+/Y control mice was *n*=6 for each tissue. At 25 weeks of age, sample sizes were as follows: brain, *n*=8 per genotype; liver, *n*=10 per genotype; quadriceps, *n*=10 per genotype; and spinal cord, *n*=8 per genotype. Note that three quadriceps samples at 25 weeks were lacking detectable SPM; thus, ratio could not be determined. Column heights indicate the means of each group with s.e.m. Statistical significance was determined individually for each polyamine, total polyamines and SPD/SPM ratio using multiple, unpaired two-tailed *t*-tests. *P*-values smaller than 0.05 were considered statistically significant and are indicated in bold on plots.

The diamine putrescine (PUT) serves as the precursor to SPD. Although PUT levels were generally low in the tissues studied, we observed a consistently increased concentration of PUT in brain tissue of SRS mice that was not detected in other tissues, including the spinal cord (Fig. S3A,B,D).

#### Behavioral phenotyping

The potential for neuromuscular deficits in the Sms^G56S^ variant mice was analyzed by measuring grip strength. Longitudinal assessment of grip strength revealed modest reductions in front-paw grip strength in the Sms^G56S^ mice compared to that in wild-type mice. This difference increased in magnitude when measuring grip strength using all paws, suggesting less contribution from the hind limbs in the SRS mice (Fig. 4A,B; Fig. S4A,B). These deficits were not evident when adjusted for body weight (Fig. S4C-F). Motor coordination was assessed as latency to fall from a rotarod apparatus. As the animals aged, Sms^G56S^ mice tended to perform better than their wild-type littermates (Fig. 4C; Fig. S4G), which may be associated with their smaller size as well as increased lean mass, both of which confound interpretation regarding neuromuscular and motor function. Regardless, male mice with the *Sms^G56S^* allele appeared to be somewhat protected from the age-related decrease in ability to stay on the rotarod that was evident in Sms^+/Y^ mice and is well documented in mice (Shoji et al., 2016).

**Fig. 4.**
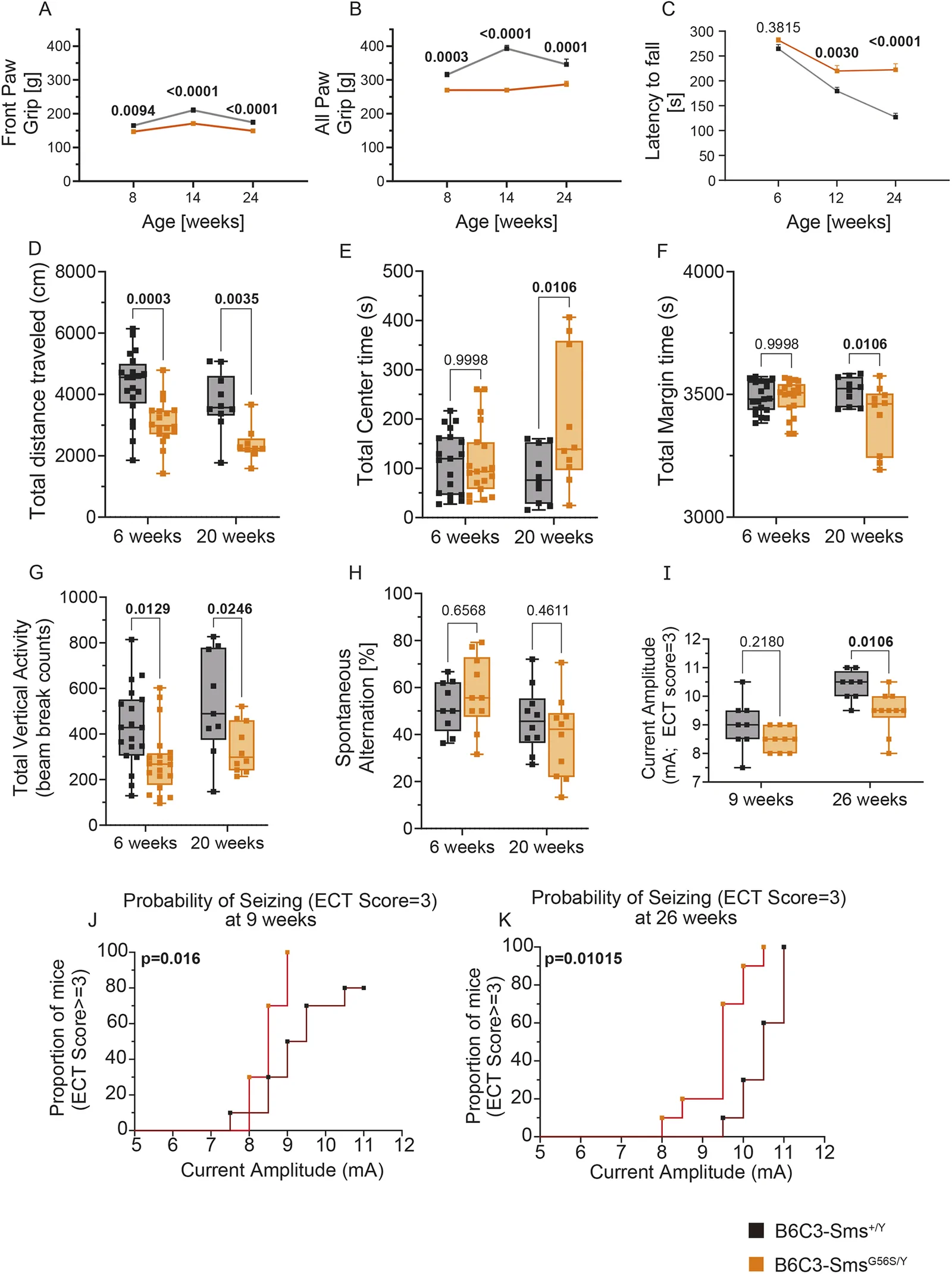
**Effects of the *Sms^G56S^* allele on behavior and seizure susceptibility.** (A,B) Neuromuscular function was assessed by measuring grip strength of forepaws (A) and all paws (B) of B6C3-Sms^+/Y^ and B6C3-Sms^G56S/Y^ mice at 8, 14 and 24 weeks of age. Data are presented as the means with s.e.m.; *n*=10 mice per genotype. (C) Motor coordination was evaluated by rotarod in B6C3-Sms^+/Y^ and B6C3-Sms^G56S/Y^ mice at 8, 14 and 24 weeks of age. Longitudinal data represent average latency to fall (in s) at each time point, with s.e.m. All statistical significance was assessed using two-way ANOVA with Šidák correction. Exact *P*-values are presented on the graph. (D-G) Overall movement and the presence of anxiety-like behaviors was assessed by open-field test in B6C3-Sms^+/Y^ and B6C3-Sms^G56S/Y^ mice at 6 and 20 weeks. Box-and-whiskers plots are representative of total distance traveled (D), total center time (E), total margin time (F) and total vertical activity (G); 6 weeks, *n*=20; 20 weeks, *n=*10. (H) Short-term memory was measured using the Y-maze in B6C3-Sms^+/Y^ (*n*=9) and B6C3-Sms^G56S/Y^ (*n*=10) mice at 6 and 20 weeks of age. Box-and-whiskers plots represent the percentage of spontaneous alternation. (I-K) Electroconvulsive threshold (ECT) was performed at 9 (*n=*10; I,J) and 26 (*n=*9; I,K) weeks in B6C3-Sms^+/Y^ and B6C3-Sms^G56S/Y^ mice. Statistical significance was assessed using two-way ANOVA with Šidák correction. Median current thresholds (mA) are depicted in box-and-whiskers plots (I). In D-I, boxes represent the 25-75th percentiles, and lines indicate the median, in individual plots. Whiskers show the minimum and maximum. Kaplan–Meier curves represent seizing probability between B6C3-Sms^+/Y^ and B6C3-Sms^G56S/Y^ mice, with comparison using Logrank (Mantel–Cox) test between the two genotypes at 9 weeks (*P*=0.016; J) and 26 weeks (*P*=0.0015; K).

B6C3-Sms^G56S/Y^ and Sms^+/Y^ mice aged 6 and 20 weeks were subjected to open-field testing for analyses of overall movement, exploratory behavior and signs of anxiety. Total distance traveled was significantly reduced in the Sms^G56S/Y^ mice at both time points, compared to that in their wild-type littermates (Fig. 4D). Although the older Sms^G56S/Y^ cohort tended to spend more time in the center of the apparatus than in the perimeter, interpretation of these results with regard to anxiety behavior is confounded by the overall reduction in movement (Fig. 4E,F). In addition to total distance traveled, Sms^G56S/Y^ mice also demonstrated reduced vertical activity, or rearing, together signifying reduced exploratory behavior compared to that of males with the wild-type *Sms* allele (Fig. 4G). Spontaneous alternation as an indicator of spatial memory was assessed using the Y-maze at ages 6 and 20 weeks. Although latency to leave the start arm did not differ between the genotypes, B6C3-Sms^G56S/Y^ mice consistently entered the arms of the ‘Y’ fewer times than wild-type males, with fewer spontaneous alternations (Fig. S5A-C). As no significant difference was evident in spontaneous alternation as a percentage of total arm entries (Fig. 4H), this analysis revealed no significant spatial-memory deficits in the B6C3-Sms^G56S^ mice – the reduction in number of spontaneous alternations is an effect of overall reduction in exploratory behavior observed in total number of arm entries and open-field analyses. The data also suggest that spontaneous alternation percentage may be declining faster with age in the B6C3-Sms^G56S/Y^ strain than in wild-type males.

#### Seizure susceptibility

Measurement of electroconvulsive threshold (ECT) was used to determine whether the *Sms^G56S^* allele affects seizure susceptibility. The minimal amplitude of current required to elicit seizure activity was reduced in B6C3-Sms^G56S/Y^ mice compared to that in wild-type males, but the difference was not statistically significant in young mice (9 weeks, Fig. 4I,J). B6C3-Sms^G56S/Y^ mice at 26 weeks of age were affected by significantly lower levels of electrostimulus than B6C3-Sms^+/Y^ mice (Fig. 4I,K). Mice on both B6 and B6C3 backgrounds were also analyzed at 40 weeks of age (Fig. S5D,E). ECT is known to increase with age. This was evident in our wild-type Sms^+/Y^ mice, most of which did not exhibit seizure activity even after 10 days of testing (defined as a seizure score ≥3 with daily exposure up to 11 mA; occurred in one of nine wild-type B6 mice and three of nine B6C3 mice). Meanwhile, ten of ten B6-Sms^G56S/Y^ and seven of ten B6C3-Sms^G56S/Y^ mice exhibited seizure activity scoring ≥3, further supporting reduced ECT in mice with the *Sms^G56S^* allele (Fig. S5D,E).

#### Alterations in serum chemical analytes

Serum was collected from B6C3-Sms^G56S/Y^ mice and their wild-type littermates at 8 and 40 weeks of age for the determination of lipids. Although low-density lipoprotein (LDL) did not significantly differ between the genotypes at 8 weeks, a highly significant reduction was evident at 40 weeks (Fig. 5A), suggesting that the age-dependent increase in LDL is absent in the SRS mice. Both high-density lipoprotein (HDL) and total cholesterol were significantly reduced in the SRS mice at both time points, again becoming highly significant with advanced age (Fig. 5B,C). Although triglycerides were reduced in young SRS mice, there was no apparent difference between genotypes in the older mice (Fig. 5D).

**Fig. 5.**
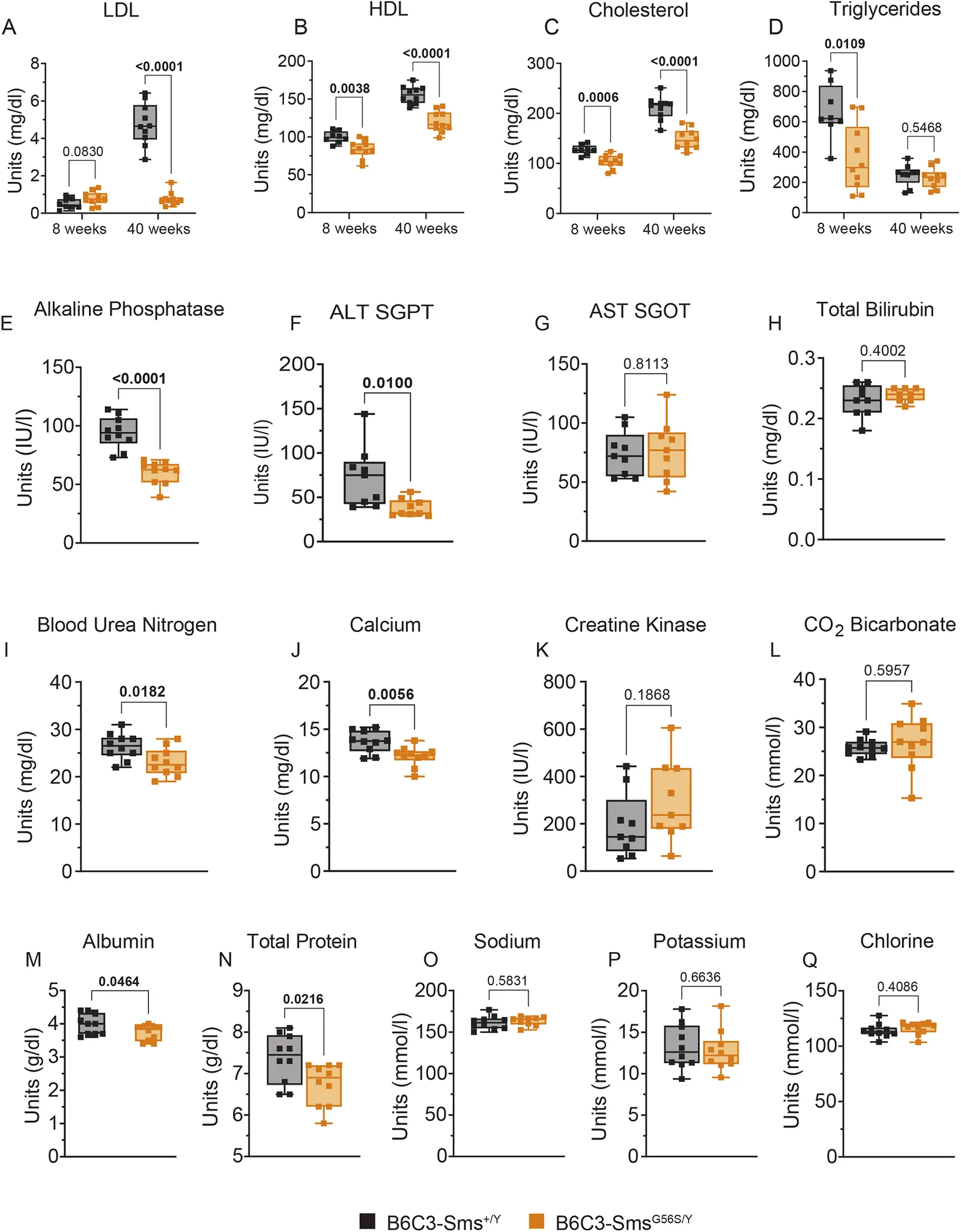
**Serum lipids are reduced and alterations in blood chemistry in Sms^G56S/Y^ mice.** (A-D) Clinical chemistry data on serum levels of low-density lipoprotein (LDL; A), high-density lipoprotein (HDL; B), cholesterol (C) and triglycerides (D) at 8 and 40 weeks of age in B6C3-Sms^G56S/Y^ and wild-type mice, expressed in mg/dl. (E-Q) Protein and chemical analytes were measured in serum of B6C3-Sms^G56S/Y^ mice and their wild-type littermates at 40 weeks of age. Liver analytes included alkaline phosphatase (E), alanine aminotransferase (ALT SGPT; F), aspartate aminotransferase (AST SGOT; G) and total bilirubin (H). Renal analytes measured included blood urea nitrogen (I), calcium (J), creatine kinase (K) and CO_2_ bicarbonate (L). Protein and chemical analytes included albumin (M), total protein (N), sodium (O), potassium (P) and chloride (Q). Parametric *t*-test was performed to evaluate differences in each analyte between B6C3-Sms^+/Y^ and B6C3-Sms^G56S/Y^ mice. Data are represented as interleaved box-and-whiskers plots, with boxes representing the 25-75th percentiles and lines indicating the median in individual plots. Whiskers show the minimum and maximum.

Serum collected at 40 weeks of age was additionally tested for altered levels of liver and kidney analytes, proteins and electrolytes. Serum of B6C3-Sms^G56S/Y^ and Sms^+/Y^ mice revealed significant reductions in alkaline phosphatase and alanine aminotransferase (ALT SGPT) in the SRS mice (Fig. 5E,F), whereas aspartate aminotransferase (AST SGOT) and total bilirubin levels were unchanged (Fig. 5G,H). Renal analytes, including blood urea nitrogen (BUN) and calcium, were also significantly reduced, whereas creatine kinase was unchanged (Fig. 5I-K). Serum proteins, including albumin and total protein, were reduced in the B6C3-Sms^G56S/Y^ mice (Fig. 5M,N). Electrolytes (sodium, potassium and chloride) and acid–base markers (CO_2_ bicarbonate) were unchanged (Fig. 5L,O-Q).

#### Brain immunohistochemistry in Sms^G56S/Y^ mice

At the completion of phenotyping studies (40 weeks of age), animals on both backgrounds were necropsied, and tissues were collected for histological analyses. Standard Hematoxylin and Eosin staining of the motor cortex, hippocampus and cerebellum revealed no obvious morphological differences between the genotypes. Additional staining was performed to visualize astrocytes and microglial activation (GFAP and IBA1, respectively) as well as LAMP1, which has been associated with SRS in a *Drosophila* model (Fig. 6A-I; Fig. S6A-I) (Li et al., 2017). Of these markers, GFAP staining was increased only in the hippocampus in the B6C3-Sms^G56S/Y^ strain, compared to that in the B6C3-Sms^+/Y^ mice (Fig. 6B,J). Mice on the pure B6 strain also displayed increased GFAP staining in the cerebellum, with a trend toward increased staining in the hippocampus (Fig. S6B,C,J). IBA1 and LAMP1 staining did not differ between the hemizygous and wild-type B6C3 strains (Fig. 6K,L). However, IBA1 staining was increased in the motor cortex and hippocampus, while LAMP1 staining was increased in the cerebellum, in B6-Sms^G56S/Y^ mice, compared to wild-type males (Fig. S6D,E,I,J). Although the differences were significant, the magnitude of these changes in positively stained cells did not exceed 5% of the total population, potentially accounting for the lack of significant differences in the more heterogeneous hybrid strain.

**Fig. 6.**
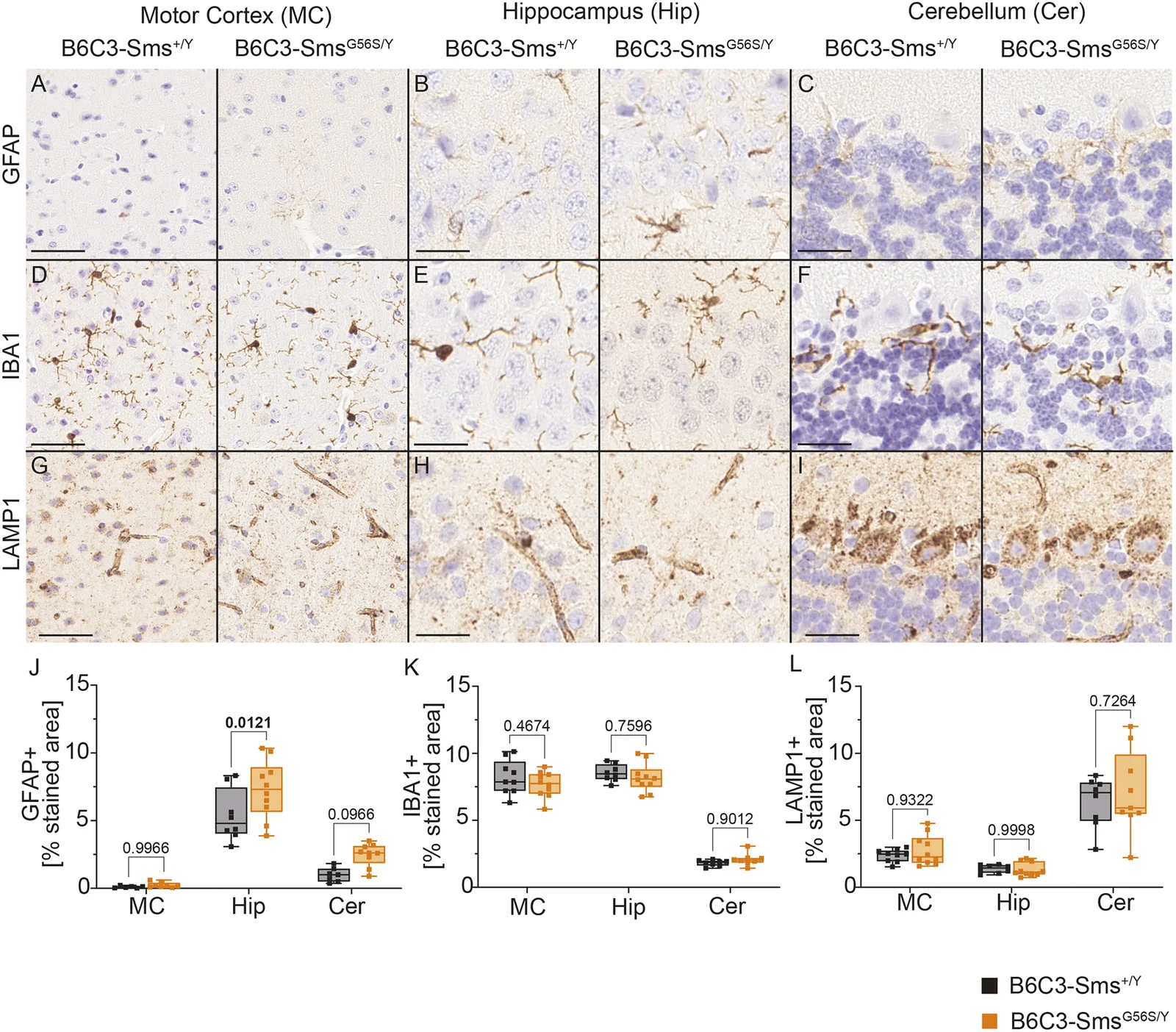
**Effects of the *Sms^G56S^* allele on regional protein expression in the brain.** (A-I) Representative GFAP, IBA1 and LAMP1 immunohistochemistry of brain regions including motor cortex (A,D,G), hippocampus (CA1) (B,E,H) and cerebellum (C,F,I) at 40 weeks in B6C3-Sms^G56S/Y^ (*n=*10) and B6C3-Sms^+/Y^ (*n=*8) mice. Motor cortex: 20×, scale bars: 25 μm. Hippocampus and cerebellum: 40×, scale bars: 50 μm. (J-L) Quantifications of GFAP (J), IBA1 (K) and LAMP1 (L) immunostaining represent the percentage of stained area in the motor cortex (A,D,G), hippocampus (B,E,H) and cerebellum (C,F,I) over total area shown. Data are represented as interleaved box-and-whiskers plots, with boxes representing the 25-75th percentiles and lines indicating the median in individual plots. Whiskers show the minimum and maximum. Multiple comparisons were performed using two-way ANOVA with Šidák correction.

## DISCUSSION

Males in the SRS population generally exhibit a range of symptoms that vary in severity (Schwartz et al., 2013) and may reflect the effect of the specific variant on enzymatic activity. The glycine residue at codon 56 of the SMS protein is evolutionally conserved across species (de Alencastro et al., 2008). The resulting G56S variant of the *SMS* gene was the second pathogenic variant to be associated with SRS and was originally described in a Brazilian family that included three severely affected males who were clinically evaluated and diagnosed with SRS, as well as two additional males who were likely to be affected (de Alencastro et al., 2008). Although this has been the sole literature report of the G56S variant in human patients, there have been six unrelated families identified with the variant (The Snyder-Robinson Foundation). In considering the applicability of our Sms^G56S/Y^ mouse models to the human condition, we compiled the available clinical characteristics of these patients to annotate consistencies (Table 2). Among individuals with the *SMS^G56S^* allele, we again observed some variability, including in patients within a single family. However, the overall severity of disease within the group appears to be more consistent than that across the SRS population. All patients with the G56S allele exhibited global developmental delay and intellectual disability, gait and speech abnormalities, dysmorphic features, seizures, osteoporosis and/or kyphoscoliosis, hypotonia and thin habitus.

**Table 2.** Clinical observations of patients with the SMS^G56S^ variant and relevance to Sms^G56S^ mouse models

	Family 1 (de Alencastro et al., 2008)			Family 2	Family 3	Family 4		Family 5	B6-Sms^G56S/Y^	B6C3-Sms^G56S/Y^
Gene variant	*SMS* (NM_004595.5), c.166G>A (p.Gly56Ser)								*Sms* (NM_009214.4), c.166_167delinsTC (p.Gly56Ser)	
Inheritance	X-linked recessive; 100% skewed XCI in mother	X-linked recessive; skewed XCI in mother	X-linked recessive; skewed XCI in mother	X-linked recessive; 100% skewed XCI in mother	X-linked recessive	X-linked recessive	X-linked recessive	*De novo*	X-linked recessive	
Sex	Male	Male	Male	Male	Male	Male	Male	Male	Male	
Pre/neonatal findings	Born at term, uneventful pregnancy; 50th percentile length, weight, head circumference	Born at term, uneventful pregnancy; 10th percentile birth weight, 50-75th percentile head circumference	Born at term, uncomplicated pregnancy/delivery; 50-75th percentile birth weight, 10-25th percentile length	Born at term; 93rd percentile length, 61st percentile weight, 75th percentile head circumference	Born at term, no birth complication; 28th percentile weight, 17th percentile head circumference	Born at term; 62nd percentile length, 6th percentile weight, 15th percentile head; precipitous delivery, NICU/respiratory distress; thrombocytopenia, hypoglycemia	Born at term; 60th percentile length, 15th percentile weight; NICU/respiratory distress; thrombocytopenia	89th percentile length, 20th percentile weight; thrombocytopenia, hypoglycemia	Reduced birth rate and high pre-weaning mortality (35% survival)	Born at Mendelian ratio; 84% survival
Age at evaluation	12 years	23 years	6 years	3 years	19 years	10 years	11 years	14 years	0-40 weeks	
Head circumference	50th percentile	50th percentile	50-98th percentile	97th percentile	Unknown	Unknown	Unknown	Unknown	N/A	
Height	<3rd percentile	Could not be evaluated (kyphoscoliosis)	<3rd percentile	>95th percentile	5th percentile	50-75th percentile	<5th percentile	<5th percentile	Reduced length	
Weight	<3rd percentile	25-50th percentile	25-50th percentile	25-50th percentile	<1st percentile	<5th percentile	<1st percentile	<1st percentile	Decreased	
Global developmental delay/intellectual disability	Yes	Yes	Yes	Yes	Yes	Yes	Yes	Yes	Reduced survival, slowed weight gain, reduced exploratory activity, lower seizure threshold	Slowed weight gain, reduced exploratory activity, reduced seizure threshold
Ambulation/age at walking	5 years, currently unable to stand alone or walk	Never walked unassisted	5 years, wide-based gait, unsteady and irregular	Non-ambulatory at 3 years	Ambulatory with increased use of wheelchair	Walks with assistance at 6 years	Non-ambulatory at 32 months	Ambulatory with increased use of wheelchair	Reduced locomotor activity	
Speech/age at first words	Nonverbal	5 years; nasal and slow speech	Nonverbal	Non-verbal at 3 years	20 months; three-word sentences at 6 years	2 years, sentences at 5 years	One to two words at 30 months	Speech delay at 9 years	N/A	
Behavior	Not reported	Not reported	Intense agitation		Behavior concerns			Behavior concerns	Increased fear response (Akinyele et al., 2024)	Reduced locomotor activity and exploratory behavior
Seizures	Yes, generalized tonic-clonic and absence	Yes, generalized	Yes, generalized	Yes, absence	Yes, Lennox-Gastaut syndrome, developmental regression with seizures	Yes, multifocal partial epilepsy	Yes, infantile spasms, Lennox-Gastaut syndrome, developmental regression with seizures	Yes	Reduced electroconvulsive threshold	
Hypotonia	Yes	Yes	Yes	Yes	Yes	Yes	Yes	Yes	Unknown	
Dysmorphic features	Mild mandibular prognathism, small upper lip and full lower lip, short philtrum, abnormal palate, abnormal ears	Mild mandibular prognathism, small upper lip and full lower lip, short philtrum, abnormal ears	Mild mandibular prognathism, small upper lip and full lower lip, short philtrum, pronounced septal deviation	Mild mandibular prognathism, small upper lip with prominent full lower lip, high arched palate	Mild mandibular prognathism, small upper lip with prominent full lower lip; normal palate (unknown: asymmetry, philtrum length, ears)	Small upper lip with prominent full lower lip, smooth philtrum, abnormal ears (unknown: prognathism, asymmetry, palate)	Small upper lip and prominent full lower lip, facial asymmetry, abnormal ears, normal palate (unknown: mandibular prognathism, philtrum length)	Small upper lip with prominent full lower lip (unknown: asymmetry, mandibular prognathism, philtrum length, palate, ears)	N/A	
Musculoskeletal features	Osteoporosis, kyphoscoliosis, camptodactyly without arachnodactyly, platyspondyly, pectus carinatum	Kyphoscoliosis, pectus carinatum, long hands, hyperextensible fingers, severe limb joint contractures; no osteoporosis evaluation but no fractures	Kyphoscoliosis, pectus carinatum, hyperextensible fingers, halluces valgi; no osteoporosis evaluation but no fractures	Osteoporosis, kyphoscoliosis, pectus excavatum, long hands and fingers (unknown: hyperextensible fingers)	Osteoporosis with multiple fractures, bone age 11 years (chronological age 18 years), kyphoscoliosis, long hands and fingers, hyperextensible fingers	Osteoporosis, pectus carinatum (unknown: kyphoscoliosis, hand or finger features)	Osteoporosis with multiple fractures, pectus excavatum, long hands and fingers (unknown: kyphoscoliosis, hyperextensible fingers)	Osteoporosis with multiple fractures kyphoscoliosis, pectus excavatum (unknown: hand or finger features)	Reduced bone mineral density by microCT (Akinyele et al., 2024); none detected by DEXA	No bone mineral density changes by DEXA; microCT not performed
Cardiology	No concerns; normal ECG and echocardiogram	No concerns; ECG normal	No concerns; ECG normal	Ventricular septal defect corrected surgically	−		Small arteriovenous fistula at birth		Unknown	
Vision	High myopia (−8, −9 dpt)	High myopia (−4.5 dpt)	Normal	No myopia, strabismus	No myopia	No myopia	No myopia	Unknown	Unknown	
Hearing	Normal	Normal	Normal	Response detectable up to 35 dB at 3 years		Passed newborn hearing screen, questionable hearing at 10 years			Response to auditory stimulation (Akinyele et al., 2024)	Unknown
Brain imaging	MRI: small bilateral pallidal calcifications; no obvious structural abnormality	C/T normal	C/T normal	Developmental venous anomaly on C/T		No abnormalities on MRI at 1 year	Mild ventriculomegaly; questionable atrophy of left hippocampus on MRI at 7 months	Slightly enlarged ventricles on MRI at 2 years	MRI: reduced total and regional (amygdala, corpus callosum, hippocampus) brain volumes (Akinyele et al., 2024)	Unknown
Other	Thin habitus with diminished muscle bulk and subcutaneous fat; ectopic right kidney	Thin habitus with diminished muscle bulk and subcutaneous fat, strength and motor coordination appear normal	Thin habitus with diminished muscle bulk but normal muscle strength	Thin habitus; Neuroblastoma 9 months; anemia at 3 years	Thin habitus	Thin habitus; family history of deceased males at 6 months and 18 years	Thin habitus; family history of deceased males at 6 months and 18 years; chalky urine with renal calculus at 9 years	Thin habitus; Crohn's disease, TPN, ileostomy; bladder sediment; anemia, thrombocytopenia, hyponatremia	Increased lean tissue percentage; decreased fat mass percentage; normal motor coordination and muscle strength	
Reproductive	Cryptorchidism			Cryptorchidism	Unilateral cryptorchidism; undetectable testosterone at 18 years			Cryptorchidism	Infertility: mating pairs set at 2 months of age (WT female×hemizygous male) fail to produce litters within 2-month mating period; viable sperm isolated, cryopreserved and able to fertilize oocytes via IVF	

C/T, computed tomography; DEXA, dual-energy X-ray absorptiometry; dpt, diopter; ECG, electrocardiogram; IVF, *in vitro* fertilization; microCT, micro-computed tomography; MRI, magnetic resonance imaging; N/A, not applicable; NICU, neonatal intensive care unit; PUT, putrescine; SMS, spermine synthase; SPD, spermidine; SPM, spermine; SRS, Snyder–Robinson syndrome; TPN, total parenteral nutrition; WT, wild type; XCI, X-chromosome inactivation.

Consistent with the thin habitus observed in patients, the most obvious manifestation of the *Sms^G56S^* allele in our mouse models is a reduction in body size. All patients with the G56S variant measured below the 50th percentile in weight, with five of the eight in the bottom fifth percentile. Sms^G56S/Y^ mice on both backgrounds weighed significantly less than their wild-type littermates, with body composition analyses revealing reduced body fat percentage, consistent with the asthenic build noted. Human patients with this variant tend to be of short stature, with five of seven patients measuring below the fifth percentile, at the time of evaluation (Table 2). Similarly, Schwartz et al. (2013) reported that 73% of all patients with SRS exhibited mild short stature, defined as 1-2 standard deviations below the mean.

Although overall lean tissue mass was decreased in our mouse models, examining it as a percentage of total body weight revealed an increase in lean mass percentage in association with the pathogenic allele. Similarly, our data indicate a reduction in grip strength in the SRS mice, although adjusting for body weight eliminated the deficit, consistent with previous results in the B6-Sms^G56S/Y^ mouse (Akinyele et al., 2024). It is notable that the modest reduction in absolute forepaw strength was amplified when strength was measured across all four paws, suggesting additional weakness in the hindlimbs of SRS mice. This is also consistent with the reduced vertical activity observed in the open-field test. Although all G56S patients display hypotonia and thin habitus, some have been reported to have normal muscle strength (de Alencastro et al., 2008). In fact, decreased functional muscle strength has not been reported in patients with SRS, despite the consistently low muscle mass reported (Schwartz et al., 2013). Additionally, the methods used for determining diminished muscle mass in the patients reported are unclear and potentially observational. Implementation of standardized reporting methods and strength testing measures for this population are needed for accurate assessment of this portion of the phenotype. Our mouse models also revealed discrepancies in age-related body composition trends. Whether this occurs in the human phenotype is unclear, with these data suggesting value for longitudinal analyses.

All eight G56S patients exhibit gait abnormalities, with some unable to walk unassisted (Table 2). In rotarod analyses, our SRS mouse models revealed no motor coordination deficits; however, the notable reduction in body size in the SRS mice confounds interpretation. Further open-field testing revealed reduced locomotor and rearing activity in Sms^G56S/Y^ mice, supporting a potential deficit in natural exploratory behavior. Although no overt gait abnormalities were apparent in our strains, additional testing will be necessary to rule out more subtle changes. Our open-field findings differed in certain behavioral parameters from those previously reported in the pure C57Bl6/J strain (Akinyele et al., 2024). Although both studies consistently demonstrated an overall reduction in locomotor activity in SMS-deficient mice, differences in center versus perimeter exploration likely reflect methodological variations in test administration, including initial mouse placement and trial duration. In the present study, mice were placed in the center of the arena and monitored over 60 min, whereas the prior study (Akinyele et al., 2024) initiated the mice near the perimeter and evaluated activity over 15 min. In both scenarios, the reduction of exploratory activity complicates an interpretation of anxiety-related behavior using this method alone. Additionally, we observed reduced locomotor activity at both 6 and 20 weeks of age, while the previous study (Akinyele et al., 2024) reported significant reductions only after 18 weeks, suggesting that our testing paradigm may be more sensitive to earlier behavioral deficits.

Consistent with the reduction in ECT observed in our mouse models, 100% of the patients with the *SMS^G56S^* variant reported seizures of various types (Table 2). Given the existing cognitive and bone-health vulnerabilities of patients with SRS, even modest seizure burden can have outsized effects. Additionally, treatment-resistant epilepsy is relatively common in SRS, likely stemming from underlying polyamine-related neurodevelopmental abnormalities (Schwartz et al., 2013). The reduced seizure threshold observed in our SMS^G56S^ mouse models directly links SMS deficiency with increased neuronal excitability, providing a platform for investigating treatment options and mechanisms of drug resistance.

Our phenotyping of the Sms^G56S^ mouse models also identified several properties that have not been thoroughly investigated in patients with SRS, providing avenues for further study. The reduction in fat mass and asthenic build of patients with SRS, combined with hypotonia and developmental delay, prompted the measurement of circulating lipid profiles in our mice (Table 2). Plasma of mice with the *Sms^G56S^* variant contained reduced levels of both LDL and HDL, as well as cholesterol and triglycerides, pointing to disrupted lipid metabolism that may contribute to the pathology or serve as a biomarker for therapeutic development. Several recent studies have linked polyamines with lipid metabolism and cholesterol biosynthesis (Cruz-Pulido et al., 2024; Pankoke et al., 2022; Yuan et al., 2025), although this has not yet been investigated in SRS. Metabolic panels were also obtained from our mouse models, as dysregulated polyamine metabolism is associated with organ damage, and polyamines are well-known regulators of ion channels, potentially influencing electrolyte balances (Zahedi et al., 2022; Nichols and Lee, 2018). The absence of elevated liver and kidney enzymes effectively rule out organ injury or toxicity in the SRS mice. Instead, the observed reductions in BUN and ALT SGPT, paired with normal AST SGOT level, likely reflect the reduction in absolute muscle mass and, consequently, limited amino acid turnover. Indeed, low ALT SGPT activity has been correlated with sarcopenia, loss of striated muscle mass and frailty in several populations, including individuals of advanced age and those with cancer (Weiss et al., 2026; Vespasiani-Gentilucci et al., 2018). Further, reductions in albumin and total protein suggest reduced hepatic protein synthesis, consistent with the reduction in ribosome-associated gene expression reported previously (Akinyele et al., 2024). That creatine kinase and AST SGOT are unchanged supports muscle atrophy driven by metabolic insufficiency, rather than overt tissue damage and membrane disruption. Finally, reductions in alkaline phosphatase and circulating calcium are consistent with the bone phenotype observed in patients with SRS and mouse models of the disorder (Akinyele et al., 2025; Koerner et al., 2024; Yorgan et al., 2024). Given the translational implications of these findings, an analysis of clinical records or samples to aggregate and confirm these metabolic trends in patients with SRS is warranted. Additionally, although hemizygous male mice failed to produce offspring when paired with wild-type females, viable sperm was isolated and capable of fertilizing oocytes via *in vitro* fertilization. In patients with SRS (G56S), cryptorchidism was reported in 50%, with undetectable testosterone measured in one patient (Table 2), suggesting a reproductive phenotype worthy of further investigation.

Patient-derived (proband) lymphoblastoid cell lines containing the G56S allele have undetectable levels of SMS enzymatic activity, resulting in SPD/SPM ratios ranging from sixfold to tenfold those of wild-type cells (de Alencastro et al., 2008; Murray-Stewart et al., 2018). Our comprehensive analyses of polyamine profiles across tissues of wild-type and Sms^G56S^ mice emphasizes the unique requirements of individual tissues in maintaining polyamine homeostasis. As an analysis of this nature is impossible in the human context, the tissue-specific effects observed in the mouse models provide insight into the biochemistry driving the primary disease sites, thereby informing treatment strategies and therapeutic windows. These measurements will also support translational developments, such as the identification of surrogate biomarkers as proxies for tissue-specific deficiencies in human patients.

On both backgrounds, male mice harboring the *Sms^G56S^* variant displayed elevation of putrescine in brain tissue compared to that in the brain tissue of wild-type mice. This appears to be a tissue-specific response, as the putrescine concentration of other tissues examined tended to be decreased or were unchanged. Similarly, the levels of putrescine in SRS patient-derived lymphoblastoid and fibroblast cell lines are less than those from age- and sex-matched unaffected donors (Murray-Stewart et al., 2018; Murray Stewart et al., 2020). Interestingly, mice with the gyro (Gy) mutation, a deletion of an X-chromosome region including the *Sms* gene, had dramatically elevated levels of putrescine specifically in the brain, while levels in the heart and liver were reduced (Mackintosh and Pegg, 2000). It is unknown whether this putrescine elevation also occurs in brain tissue of patients with SRS or whether the change contributes to the pathology or serves a protective role.

Overall, we conclude that the outcrossed B6C3F1.*Sms^G56S^* murine SRS mouse model recapitulates the phenotype observed in the pure C57Bl/6J strain, with the added benefit of efficient offspring generation at the expected Mendelian ratios. It should be noted that some aspects of the phenotype, including body mass reduction, seizure susceptibility and markers of neuroinflammation, appear less severe in the hybrid strain than in the pure C57Bl/6J strain. However, the diversion from wild type remains measurable and is offset by the dramatic improvement in viability, which greatly facilitates the procurement of experimental cohorts that are of sufficient power for statistical analyses. Importantly, the defined phenotype is consistent with several aspects of SRS in human patients, including asthenic build, seizure susceptibility, reduced locomotor activity and reduced fertility. Finally, our comprehensive catalog of altered polyamine biochemistry across SRS mouse tissues provides a valuable resource that validates the model and informs translational strategies using data that cannot be obtained from human tissue.

## MATERIALS AND METHODS

### Mouse statement

All animal experiments and procedures adhere to the Office of Laboratory Animal Welfare guidelines and were approved by the Institutional Animal Care and Use Committee at The Jackson Laboratory. All mice were maintained in a specific pathogen-free research colony at The Jackson Laboratory, in individually HEPA-filtered polysulfonate cages, with controlled conditions including 12-h light/dark cycles (06:00 to 18:00), room temperature of 22±4°C with 50±15% humidity, and 15 air exchanges/h. Animals had access to acidified tap water (pH 2.5-3.0) and normal rodent chow *ad libitum*. Animals were checked weekly for welfare, and individuals presenting more than 15% of maximum body weight loss were considered to be at humane endpoint and euthanized.

### Mouse generation and colony establishment

Using CRISPR/Cas9-targeted mutagenesis methodologies, we generated the G56S variant allele of the murine *Sms* gene in the C57BL/6J inbred mouse strain. Briefly, exon 2 of the *Sms* locus on chromosome X was targeted with sgRNAs designed to substitute the wild-type GGC codon at position 56 with TCC, thereby replacing a glycine residue with serine (*Sms^G56S^*). The Cas9 endonuclease, these sgRNAs and an oligonucleotide containing the G56S variant were electroporated into single-cell zygotes derived from C57BL/6J mice (JAX strain #000664), and embryos were transferred to pseudopregnant females. Correct targeting was confirmed in the resulting pups by Sanger sequencing (Fig. 1B), which also revealed the formation of an EcoRI restriction enzyme site in the *Sms^G56S^* allele that is absent from the wild-type allele.

Of several founders containing the G56S variant, one male was found to transmit the desired allele to females when mated with the inbred strain. The subsequent mating of these heterozygous carrier females with the inbred strain generated an expanded population of *B6-Sms^G56S^* mutant mice (JAX strain #031170; C57BL/6J-*Sms^em2Lutzy/J^*; RRID: IMSR_JAX:031170). To produce animals on a hybrid genetic background, heterozygous females from this C57BL/6J strain (JAX strain #031170) were intercrossed with C3H/HeJ males (JAX strain #000659) to establish the B6C3F1-*Sms^em2Lutzy^/J* (B6C3-Sms^G56S^) strain (JAX strain #033707; RRID: IMSR_JAX:033707). For colony maintenance, B6C3F1.*Sms^G56S^* heterozygous females were crossed with B6C3F1/J males at every generation (JAX strain #100010).

### Genotyping

Pups were sampled at P0 by toe clipping and genotyped by quantitative PCR using the following primers and fluorescent probe sets. For sex determination: mutant forward (5′-AAAGGAATTCTGGAGGCTGG-3′), mutant reverse (5′-TCTTTGGAGTGAATGTCTGCTC-3′), wild-type forward (5′-GACTAGGTTCATAGGCACTGG-3′), wild-type reverse (5′-CCGCCAAAACTCCTTCTCTAC-3′), probe-mutant-FAM (5′-AGCAGCATTGAGGAGGATGTGACAG-3′) and probe-wild-type-HEX (5′-CCCCAGATGGTACCCACAGAACTTG-3′). For *Sms* genotyping: forward (5′-TGGCAGGACCATGGATATTT-3′), reverse (5′-CCTAGTCCCAGCTGTGTTCA-3′), probe-mutant (5′-CACGAACAAGAATTCCAGGTAAG-3′) and probe-wild type (5′-CACGAACAAGAATGGCAGGT-3′).

### Western blot analysis of SMS

Brain, gonad and skeletal muscle lysates from male wild-type (B6-SMS^+/Y^) and hemizygote (B6-Sms^G56S/Y^), along with female wild-type (B6-Sms^+/X^) and heterozygote (B6-Sms^G56S/X^), mice were prepared in RIPA buffer (Thermo Fisher Scientific, 89901) containing Halt™ Protease Inhibitor Cocktail (Thermo Fisher Scientific, 1861284). Total protein content was quantified by detergent compatible assay (Bio-Rad, 5000113, 5000114, 5000115) and adjusted to 5 mg/ml in RIPA buffer. Protein lysates were prepared for automated western blotting using the Simple Western Wes system (ProteinSimple, 004-600). In short, samples were mixed at a 4:1 ratio with 5× fluorescent master mix, producing a final protein concentration of 0.80 mg/ml and heated at 95°C for 5min. Samples plus biotinylated molecular mass standards (ProteinSimple, PS-ST01EZ-8) were loaded into individual capillaries along with blocking solution, biotin labeling reagent, wash buffers, primary antibodies, horseradish peroxidase-conjugated secondary antibodies and chemiluminescent substrate into a plate prefilled with stacking and separation matrices (ProteinSimple, SM-W001).

For immunodetection, anti-SMS antibody was used at a dilution of 1:200 (Abcam, ab156879) with a 30-min incubation time. Chemiluminescent signal was captured, and the resulting image was analyzed using the Compass for Simple Western software package (Compass for SW, version 6.2.0). Total protein staining was performed following ProteinSimple instructions. The SMS peak area was normalized to total protein area following ProteinSimple recommendations and standard guidelines. Relative fold change was calculated as the ratio of experimental sample corrected area to the corresponding reference sex group corrected area (B6-Sms^+/X^ for females or B6-Sms^+/Y^ for males); *n=*4-6 mice, as biological replicates.

### Body weight and composition

Animals were weighed biweekly (*n*=10 per cohort) to monitor growth and weight gain. Body composition, including tissue and bone parameters, was measured using DEXA in B6-Sms^+/Y^ and B6-Sms^G56S/Y^ mice, as well as the mixed-background B6C3-Sms^+/Y^ and B6C3-Sms^G56S/Y^ mice, at 6, 12 and 20 weeks. Following intraperitoneal injection of a 2% ketamine/xylazine mixture (0.05-0.1 ml/10 g body weight) to induce anesthesia, the mouse is positioned on the exposure platform of the apparatus (PIXImus) or on a disposable plastic specimen tray in the prone position with limbs lateral to avoid superior–inferior shadowing. Scans were conducted over 4 min, and data were converted to obtain bone parameters (bone mineral density, bone mineral content, bone area), lean mass and fat mass; *n=*3-10.

### Polyamine quantification

Lysates were prepared from frozen tissues (brain, spinal cord, quadriceps and liver) in cold 0.083 M glycine buffer (pH 8) using a handheld tissue homogenizer. Aliquots of each sample were acid extracted and centrifuged at 14,000 ***g***, 4°C, for 10 min. Polyamines in the supernatant were labelled with dansyl chloride (Sigma-Aldrich), and intracellular polyamine concentrations were measured using an adaptation of the reverse-phase high-performance liquid chromatography method of Kabra et al., with 1,7-diaminoheptane as an internal standard (Kabra et al., 1986; Nwafor et al., 2025). Values are presented relative to total cellular protein in the same lysate, which was quantified using the Bio-Rad Protein Assay with interpolation on a bovine serum albumin standard curve. Putrescine, spermidine, spermine and diaminoheptane for use as standards were purchased from Sigma-Aldrich.

### Grip strength

Neuromuscular function was assessed via the grip strength task, using a Chatillon-Ametek Digital Force Gauge, DFIS-2 (Columbus Instruments, Chatillon-Ametek Digital Force Gauge, DFE II), in B6-Sms^+/Y^ and B6-Sms^G56S/Y^ mice at 10, 12 and 24 weeks; and B6C3-Sms^+/Y^ and B6C3-Sms^G56S/Y^ mice at 8, 14 and 24 weeks. The mouse was raised toward a T-bar portion of a metal force transducer and allowed to instinctively grasp it with its forelimbs or all limbs. The animal was then pulled downward, and peak force (in kg) at the point of release was recorded over three consecutive trials. An average of three trials, with and without body weight normalization, served as the final score. *n=*10-15.

### Rotarod

Motor coordination was evaluated by rotarod analysis using an Ugo Basile Accelerating Rotarod (Ugo Basile, model 47600) in B6-Sms^+/Y^ and B6-SMS^G56S/Y^ mice at 8, 12 and 24 weeks, and in B6C3-Sms^+/Y^ and B6C3-Sms^G56S/Y^ mice at 6, 12 and 24 weeks. After an acclimatization time of 60 min, mice were placed on the rotarod starting with one mode and reaching three different modes: (1) stationary, (2) moving at a steady 4 rpm and (3) accelerating by 0.1 rpm/s for 5 min. The test consisted of 2 days (learning and testing phases), four times each day, with a 1-min rest inter-trial. Times for the final three consecutive trials on the second day were recorded, and average latency to fall was determined for each. *n=*10-15 mice/genotype.

### Open-field assay

General activity, locomotor activity and exploratory behavior activity were assessed via the open-field task (van den Buuse, 2010) at 6 and 20 weeks of age in groups of B6C3-Sms^+/Y^ and B6C3-Sms^G56S/Y^ mice. Mice were placed in the testing room for acclimatization for 60 min, after which they were placed individually in a 40 cm×40 cm×40 cm box. Lighting was indirect via mounted LED lights. Activity was recorded over a 60-min period using tracking software (Fusion, from OmniTech Electronics™) that converts beam breaks into variables including distance traveled (cm), vertical activity and time in different areas. Body motion evaluated included time spent in the center and distance moved in the apparatus; 6 weeks, *n=*20; 20 weeks, *n=*10.

### Spontaneous alternation (Y-maze)

Short-term memory was measured using the Y-maze task with three arm lengths sized 25-35 cm, an arm lane width of 5-6 cm and a wall height of 12-18 cm. This task was performed on B6C3-Sms^+/Y^ and B6C3-Sms^G56S/Y^ mice at 6 and 20 weeks of age. Mice were habituated to the testing room for 60 min prior to testing, then placed in the start arm (A) facing the center of the Y for a 10-min test period. One arm, the ‘novel’ arm, is blocked off with a black insert. This ‘novel’ arm is alternatively chosen in B or C following a pseudorandom order when testing each mouse. After a delay of 10-30 min, the insert is removed, and the mouse is placed back in the same arena for 5 min. Movement of the mouse was recorded by a camera mounted above the maze and interfaced with tracking software (Noldus Ethovision; www.noldus.com) to evaluate spontaneous alternation; *n=*10*.*

### ECT

ECT measures the level of intensity required for an electrical current to induce minimal clonic (forebrain), maximal tonic (hindbrain/brain stem) and psychomotor (limbic) seizures in mice using an electrostimulator (Pulse Generator, #57800-001; UGO BASILE Biological Research Apparatus, Stoelting, USA). This task was performed at 9 and 26 weeks of age in B6C3-Sms^+/Y^ and B6C3-Sms^G56S/Y^ mice, as well as at 40 weeks of age in both strains, to determine potential differences in seizure susceptibility between SRS mice and wild-type littermates. Animals were subjected to a short electrical stimulus (0.2 s, 299 Hz, 1.6 ms pulse width) starting at 5 mA, followed by release onto its cage bedding and observation for seizure activity based on a defined scale (range, 0-5). Retesting occurred at 24-h increments (no more than one shock per day), increasing the stimulus by 0.5 mA intervals, until a seizure scale score ≥3 was recorded [prolonged jaw and forelimb clonus, ventral or dorsal neck flexion, loss of posture, tonic extension of forelimbs and/or hindlimbs (90° angle to torso), lasting several seconds] (Frankel et al., 2001). Data are expressed as the mA threshold at which a level 3 seizure occurs. If a mouse did not reach a level 3 seizure after 10 test days, the experiment was discontinued. Those mice experiencing a seizure lasting more than 1 min were euthanized, regardless of the level of the seizure on the scale; 9 weeks, *n=*8; 26 weeks, *n=*20; 40 weeks, *n=*9-10.

### Serum collection and clinical chemistry

Blood was collected via cardiac puncture on CO_2_-euthanized mice in a serum microtainer^®^ (Becton-Dickinson, #365957) and held at room temperature for 30 min. The collected blood was centrifuged at 14,000 ***g*** at 4°C for 10 min, and the serum was separated from the whole blood and stored at −80°C until further use. Serum was analyzed for albumin, alkaline phosphatase, ALT SGPT, AST SGOT, BUN, calcium, creatine kinase, direct bilirubin, LDL, HDL, cholesterol, total protein, triglycerides, sodium, potassium, chloride and CO_2_ bicarbonate using a Beckman Coulter DxC 700 AU Chemistry Analyzer with associated system reagents.

### Histology

Tissues were prepared for paraffin embedding after being fixed in 10% neutral-buffered formalin for 24 to 48 h. Sagittal brain sections with a thickness of 5 μm were obtained using a Leica Bond auto-staining system (Leica Biosystems). Anti-LAMP1 (Invitrogen, 14-1071-82; 1:1000), anti-GFAP (Abcam, ab16997, RRID:AB_443592; 1:100) and anti-IBA1 (Abcam, ab178846, RRID:AB_2636859; 1:2000) primary antibodies were used for immunohistological staining with counterstaining by Mayers Hematoxylin (Fisher Scientific, 72804); *n=*10*.*

Stained slides were digitalized at 40× magnification in a Hamamatsu Nanozoomer S210 (Hamamatsu). Two regions of interest (ROIs) were chosen for the motor cortex at 20×, and the cerebellum and CA1 (hippocampal region) at 40×. ROIs were imported into CellProfiler (version 4.2.4), a free open-source program, and then analyzed via a supplemental pipeline to determine the overall stained area occupied by IBA-1, GFAP and LAMP1 staining in the total ROI area. The analysis was conducted in a double-anonymized manner to avoid bias. All statistical significance was assessed using two-way ANOVA with Šidák correction. Exact *P*-values are presented on graphs.

### Statistical analyses

Statistical analyses and graphing were performed using GraphPad Prism version 10.6.0. Statistical significance between wild-type and Sms^G56S^ strains was determined as indicated in figure legends. *P*-values smaller than 0.05 were considered statistically significant and are indicated in bold along with comparisons on plots.

## Supplementary Material

10.1242/dmm.052953_sup1 Supplementary information

## References

[DMM052953C1] Akinyele, O., Munir, A., Johnson, M. A., Perez, M. S., Gao, Y., Foley, J. R., Nwafor, A., Wu, Y., Murray-Stewart, T., Casero, R. A. Jr. et al. (2024). Impaired polyamine metabolism causes behavioral and neuroanatomical defects in a mouse model of Snyder-Robinson syndrome. *Dis. Model. Mech.* 17, dmm050639. 10.1242/dmm.050639 PMC11103582 38463005

[DMM052953C2] Akinyele, O., Tran, K. B., Johnson, M. A. and Kemaladewi, D. U. (2025). Gene replacement therapy to restore polyamine metabolism in a Snyder-Robinson syndrome mouse model. *Methods Enzymol.* 715, 271-292. 10.1016/bs.mie.2025.01.068 40382143

[DMM052953C3] Albert, J. S., Bhattacharyya, N., Wolfe, L. A., Bone, W. P., Maduro, V., Accardi, J., Adams, D. R., Schwartz, C. E., Norris, J., Wood, T. et al. (2015). Impaired osteoblast and osteoclast function characterize the osteoporosis of Snyder - Robinson syndrome. *Orphanet J. Rare Dis.* 10, 27. 10.1186/s13023-015-0235-8 PMC4428506 25888122

[DMM052953C4] Arena, J. F., Schwartz, C., Ouzts, L., Stevenson, R., Miller, M., Garza, J., Nance, M. and Lubs, H. (1996). X-linked mental retardation with thin habitus, osteoporosis, and kyphoscoliosis: linkage to Xp21.3-p22.12. *Am. J. Med. Genet.* 64, 50-58. 10.1002/(SICI)1096-8628(19960712)64:1<50::AID-AJMG7>3.0.CO;2-V 8826448

[DMM052953C5] Becerra-Solano, L. E., Butler, J., Castaneda-Cisneros, G., McCloskey, D. E., Wang, X., Pegg, A. E., Schwartz, C. E., Sanchez-Corona, J. and Garcia-Ortiz, J. E. (2009). A missense mutation, p.V132G, in the X-linked spermine synthase gene (SMS) causes Snyder-Robinson syndrome. *Am. J. Med. Genet. A* 149A, 328-335. 10.1002/ajmg.a.32641 PMC2653108 19206178

[DMM052953C6] Cason, A. L., Ikeguchi, Y., Skinner, C., Wood, T. C., Holden, K. R., Lubs, H. A., Martinez, F., Simensen, R. J., Stevenson, R. E., Pegg, A. E. et al. (2003). X-linked spermine synthase gene (SMS) defect: the first polyamine deficiency syndrome. *Eur. J. Hum. Genet.* 11, 937-944. 10.1038/sj.ejhg.5201072 14508504

[DMM052953C7] Civit, A., Ronce, N., Cogne, B., Besnard, T., Laurenceau, D., Hubert, C., Moizard, M. P., Gueguen, P., Toutain, A. and Vuillaume, M. L. (2025). Identification of a rare branch point variant in the SMS gene in a large family with a severe form of Snyder-Robinson Syndrome. *Clin. Genet.* 107, 231-233. 10.1111/cge.14643 PMC11725555 39523020

[DMM052953C8] Cruz-Pulido, Y. E., Lomascolo, N. J., May, D., Hatahet, J., Thomas, C. E., Chu, A. K. W., Stacey, S. P., Villanueva Guzman, M. D. M., Aubert, G. and Mounce, B. C. (2024). Polyamines mediate cellular energetics and lipid metabolism through mitochondrial respiration to facilitate virus replication. *PLoS Pathog.* 20, e1012711. 10.1371/journal.ppat.1012711 PMC11611256 39556649

[DMM052953C9] De Alencastro, G., McCloskey, D. E., Kliemann, S. E., Maranduba, C. M., Pegg, A. E., Wang, X., Bertola, D. R., Schwartz, C. E., Passos-Bueno, M. R. and Sertie, A. L. (2008). New SMS mutation leads to a striking reduction in spermine synthase protein function and a severe form of Snyder-Robinson X-linked recessive mental retardation syndrome. *J. Med. Genet.* 45, 539-543. 10.1136/jmg.2007.056713 18550699

[DMM052953C10] Dontaine, P., Kottos, E., Dassonville, M., Balasel, O., Catros, V., Soblet, J., Perlot, P. and Vilain, C. (2021). Digestive involvement in a severe form of Snyder-Robinson syndrome: possible expansion of the phenotype. *Eur. J. Med. Genet.* 64, 104097. 10.1016/j.ejmg.2020.104097 33186760

[DMM052953C11] Frankel, W. N., Taylor, L., Beyer, B., Tempel, B. L. and White, H. S. (2001). Electroconvulsive thresholds of inbred mouse strains. *Genomics* 74, 306-312. 10.1006/geno.2001.6564 11414758

[DMM052953C12] Kabra, P. M., Lee, H. K., Lubich, W. P. and Marton, L. J. (1986). Solid-phase extraction and determination of dansyl derivatives of unconjugated and acetylated polyamines by reversed-phase liquid chromatography: improved separation systems for polyamines in cerebrospinal fluid, urine and tissue. *J. Chromatogr.* 380, 19-32. 10.1016/S0378-4347(00)83621-X 3745383

[DMM052953C13] Koerner, T. L., Green, A. M., Pace-Farr, D. J., Zeitler, C. M., Schwartz, M. B. and Kutler, M. J. F. (2024). Bone manifestations in Snyder-Robinson syndrome. *Rare* 2, 100025. 10.1016/j.rare.2024.100025

[DMM052953C14] Larcher, L., Norris, J. W., Lejeune, E., Buratti, J., Mignot, C., Garel, C., Keren, B., Schwartz, C. E. and Whalen, S. (2020). The complete loss of function of the SMS gene results in a severe form of Snyder-Robinson syndrome. *Eur. J. Med. Genet.* 63, 103777. 10.1016/j.ejmg.2019.103777 31580924

[DMM052953C15] Leung, M., Sanchez-Castillo, M., Belnap, N., Naymik, M., Bonfitto, A., Sloan, J., Hassett, K., Jepsen, W. M., Sankaramoorthy, A., Stewart, T. M. et al. (2024). Snyder-Robinson syndrome presenting with learning disability, epilepsy, and osteoporosis: a novel SMS gene variant. *Rare* 2, 100017. 10.1016/j.rare.2023.100017 PMC11105150 38770537

[DMM052953C16] Li, C., Brazill, J. M., Liu, S., Bello, C., Zhu, Y., Morimoto, M., Cascio, L., Pauly, R., Diaz-Perez, Z., Malicdan, M. C. V. et al. (2017). Spermine synthase deficiency causes lysosomal dysfunction and oxidative stress in models of Snyder-Robinson syndrome. *Nat. Commun.* 8, 1257. 10.1038/s41467-017-01289-7 PMC5668419 29097652

[DMM052953C17] Mackintosh, C. A. and Pegg, A. E. (2000). Effect of spermine synthase deficiency on polyamine biosynthesis and content in mice and embryonic fibroblasts, and the sensitivity of fibroblasts to 1,3-bis-(2-chloroethyl)-N-nitrosourea. *Biochem. J.* 351, 439-447. 10.1042/bj3510439 PMC1221380 11023830

[DMM052953C18] Murray-Stewart, T., Dunworth, M., Foley, J. R., Schwartz, C. E. and Casero, R. A. Jr. (2018). Polyamine homeostasis in Snyder-Robinson syndrome. *Med. Sci.* 6, E112. 10.3390/medsci6040112 PMC6318755 30544565

[DMM052953C19] Nichols, C. G. and Lee, S.-J. (2018). Polyamines and potassium channels: a 25-year romance. *J. Biol. Chem.* 293, 18779-18788. 10.1074/jbc.TM118.003344 PMC6290165 30333230

[DMM052953C20] Nwafor, A., Stewart, T. M. and Casero, R. A. (2025). An enhanced method for the determination of polyamine content in biological samples by dansyl chloride derivatization and HPLC. *Methods Enzymol.* 715, 323-335. 10.1016/bs.mie.2025.01.066 PMC12683670 40382146

[DMM052953C21] Pankoke, S., Pfarrer, C., Glage, S., Muhlfeld, C. and Schipke, J. (2022). Oral supplementation with the polyamine spermidine affects hepatic but not pulmonary lipid metabolism in lean but not obese mice. *Nutrients* 14, 4318. 10.3390/nu14204318 PMC9611404 36297003

[DMM052953C22] Peron, A., Spaccini, L., Norris, J., Bova, S. M., Selicorni, A., Weber, G., Wood, T., Schwartz, C. E. and Mastrangelo, M. (2013). Snyder-Robinson syndrome: a novel nonsense mutation in spermine synthase and expansion of the phenotype. *Am. J. Med. Genet. A* 161A, 2316-2320. 10.1002/ajmg.a.36116 23897707

[DMM052953C23] Qazi, T. J., Wu, Q., Aierken, A., Lu, D., Bukhari, I., Hussain, H. M. J., Yang, J., Mir, A. and Qing, H. (2020). Whole-exome sequencing identifies a novel mutation in spermine synthase gene (SMS) associated with Snyder-Robinson Syndrome. *BMC Med. Genet.* 21, 168. 10.1186/s12881-020-01095-x PMC7446199 32838743

[DMM052953C24] Schwartz, C. E., Wang, X., Stevenson, R. E. and Pegg, A. E. (2011). Spermine synthase deficiency resulting in X-linked intellectual disability (Snyder-Robinson syndrome). *Methods Mol. Biol.* 720, 437-445. 10.1007/978-1-61779-034-8_28 21318891

[DMM052953C25] Schwartz, C. E., Bupp, C., Kutler, M. J. and Peron, A. (2013). Snyder-Robinson syndrome. In *GeneReviews((R))* (ed. M. P. Adam, J. Feldman, G. M. Mirzaa, R. A. Pagon, S. E. Wallace and A. Amemiya). Seattle, WA: University of Washington, Seattle, 1993-2026. 23805436

[DMM052953C39] Shoji, H., Takao, K., Hattori, S. and Miyakawa, T. (2016). Age-related changes in behavior in C57BL/6J mice from young adulthood to middle age. *Mol. Brain.* 9, 11. 10.1186/s13041-016-0191-9 PMC4730600 26822304

[DMM052953C26] Snyder, R. D. and Robinson, A. (1969). Recessive sex-linked mental retardation in the absence of other recognizable abnormalities. Report of a family. *Clin. Pediatr.* 8, 669-674. 10.1177/000992286900801114 5823961

[DMM052953C27] Stewart, T. M., Khomutov, M., Foley, J. R., Guo, X., Holbert, C. E., Dunston, T. T., Schwartz, C. E., Gabrielson, K., Khomutov, A. and Casero, R. A. Jr. (2020). (R,R)-1,12-Dimethylspermine can mitigate abnormal spermidine accumulation in Snyder-Robinson syndrome. *J. Biol. Chem.* 295, 3247-3256. 10.1074/jbc.RA119.011572 PMC7062180 31996374

[DMM052953C28] Stewart, T. M., Foley, J. R., Holbert, C. E., Khomutov, M., Rastkari, N., Tao, X., Khomutov, A. R., Zhai, R. G. and Casero, R. A. Jr. (2023). Difluoromethylornithine rebalances aberrant polyamine ratios in Snyder-Robinson syndrome. *EMBO Mol. Med.* 15, e17833. 10.15252/emmm.202317833 PMC10630878 37702369

[DMM052953C29] Tao, X. and Zhai, R. G. (2025). Development and characterization of a Drosophila model of Snyder-Robinson syndrome. *Methods Enzymol.* 715, 241-256. 10.1016/bs.mie.2025.01.077 PMC13196596 40382141

[DMM052953C30] Van Den Buuse, M. (2010). Modeling the positive symptoms of schizophrenia in genetically modified mice: pharmacology and methodology aspects. *Schizophr. Bull.* 36, 246-270. 10.1093/schbul/sbp132 PMC2833124 19900963

[DMM052953C31] Vespasiani-Gentilucci, U., De Vincentis, A., Ferrucci, L., Bandinelli, S., Antonelli Incalzi, R. and Picardi, A. (2018). Low alanine aminotransferase levels in the elderly population: frailty, disability, sarcopenia, and reduced survival. *J. Gerontol. A Biol. Sci. Med. Sci.* 73, 925-930. 10.1093/gerona/glx126 PMC6001897 28633440

[DMM052953C32] Wang, X. and Pegg, A. E. (2011). Use of (Gyro) Gy and spermine synthase transgenic mice to study functions of spermine. *Methods Mol. Biol.* 720, 159-170. 10.1007/978-1-61779-034-8_9 21318872

[DMM052953C33] Wang, X., Ikeguchi, Y., McCloskey, D. E., Nelson, P. and Pegg, A. E. (2004). Spermine synthesis is required for normal viability, growth, and fertility in the mouse. *J. Biol. Chem.* 279, 51370-51375. 10.1074/jbc.M410471200 15459188

[DMM052953C34] Weiss, Y. T., Oren, K., Sharon, N. B. M., Kerman, T., Hazan, I., Marom, T., Ziv, O., Segal, G. and Cohen, O. (2026). Low alanine aminotransferase blood activity, a biomarker of sarcopenia and frailty, is associated with worse post-total laryngectomy clinical outcomes. A retrospective analysis of 427 patients. *Clin. Otolaryngol.* 51, 307-312. 10.1111/coa.70047 PMC12869002 41327955

[DMM052953C35] Yorgan, T. A., Zhu, Y., Wiedemann, P., Schoneck, K., Pohl, S., Schweizer, M., Amling, M., Barvencik, F., Oheim, R. and Schinke, T. (2024). Inactivation of spermine synthase in mice causes osteopenia due to reduced osteoblast activity. *J. Bone Miner. Res.* 39, 1606-1620. 10.1093/jbmr/zjae156 39331754

[DMM052953C36] Yuan, X., Mao, Y., Chen, Y., Zhuang, J., Chen, D. and Li, X. (2025). Spermine enhances cholesterol effluence of macrophages through caspase 2-mediated autophagy. *Discov. Med.* 37, 884-893. 10.24976/Discov.Med.202537196.78 40415363

[DMM052953C37] Zahedi, K., Barone, S. and Soleimani, M. (2022). Polyamines and their metabolism: from the maintenance of physiological homeostasis to the mediation of disease. *Med. Sci.* 10, 38. 10.3390/medsci10030038 PMC9326668 35893120

[DMM052953C38] Zhang, Z., Norris, J., Kalscheuer, V., Wood, T., Wang, L., Schwartz, C., Alexov, E. and Van Esch, H. (2013). A Y328C missense mutation in spermine synthase causes a mild form of Snyder-Robinson syndrome. *Hum. Mol. Genet.* 22, 3789-3797. 10.1093/hmg/ddt229 PMC3749864 23696453

