# Supplementary material for: An improved Sms p.Gly56Ser model of Snyder–Robinson syndrome reveals phenotypic parallels with clinical features
Source: Dis Model Mech. 2026 Sep 23;19(9):dmm052953. doi: 10.1242/dmm.052953 (PMC13651952; doi:10.1242/dmm.052953)
Supplement: Supplementary file 1 [file dmm-19-052953-s1.pdf]

A

```
Mouse  MAAARHSTLDFKLGAKADGEAILKGLQSIFQEQGMTESVHTWQDHGYLATYTNKNGSFANLRIYPHGLVLLDLQSYDSDVQGKQETDSLL
       ::::::::::: ::::::::.::::::::::::::.::::::::::::::::::::::::::::::::::::::::::.:.:::.: :.:
Human  MAAARHSTLDFMLGAKADGETILKGLQSIFQEQGMAESVHTWQDHGYLATYTNKNGSFANLRIYPHGLVLLDLQSYDGDAQGKEEIDSIL
                10        20        30        40        50        60        70        80        90

              100       110       120       130       140       150       160       170       180
Mouse  NKIEEKMKELSQDSTGRVKRLPPIVRGGAIDRYWPTADGRLVEYDIDEVVYDEDSPYQNIKILHSKQFGNILILSGDVNLAESDLAYTRA
       ::.::.::::::::::::::::::::::::::::::::::::::::::::::::::::::::::::::::::::::::::::::::::::
Human  NKVEERMKELSQDSTGRVKRLPPIVRGGAIDRYWPTADGRLVEYDIDEVVYDEDSPYQNIKILHSKQFGNILILSGDVNLAESDLAYTRA
              100       110       120       130       140       150       160       170       180

              190       200       210       220       230       240       250       260       270
Mouse  IMGSGKEDYTGKDVLILGGGDGGILCEIVKLKPKMVTMVEIDQMVIDGCKKYMRRTCGDVLDNLRGDCYQVLIEDCIPVLKMYAKEGREF
       ::::::::::::::::::::::::::::::::::::::::::::::::::::::.:::::::::.:::::::::::::::: ::::::::
Human  IMGSGKEDYTGKDVLILGGGDGGILCEIVKLKPKMVTMVEIDQMVIDGCKKYMRKTCGDVLDNLKGDCYQVLIEDCIPVLKRYAKEGREF
              190       200       210       220       230       240       250       260       270

              280       290       300       310       320       330       340       350       360
Mouse  DYVINDLTAVPISTSPEEDSTWDFLRLILDLSMKVLKQDGKYFTQGNCVNLTEALSLYEEQLGRLYCPVEFSKEIVCVPSYLELWVFYTV
       ::::::::::::::::::::::.::::::::::::::::::::::::::::::::::::::::::::::::::::::::::::::::::
Human  DYVINDLTAVPISTSPEEDSTWEFLRLILDLSMKVLKQDGKYFTQGNCVNLTEALSLYEEQLGRLYCPVEFSKEIVCVPSYLELWVFYTV
              280       290       300       310       320       330       340       350       360

Mouse  WKKAKP
       ::::::
Human  WKKAKP
```

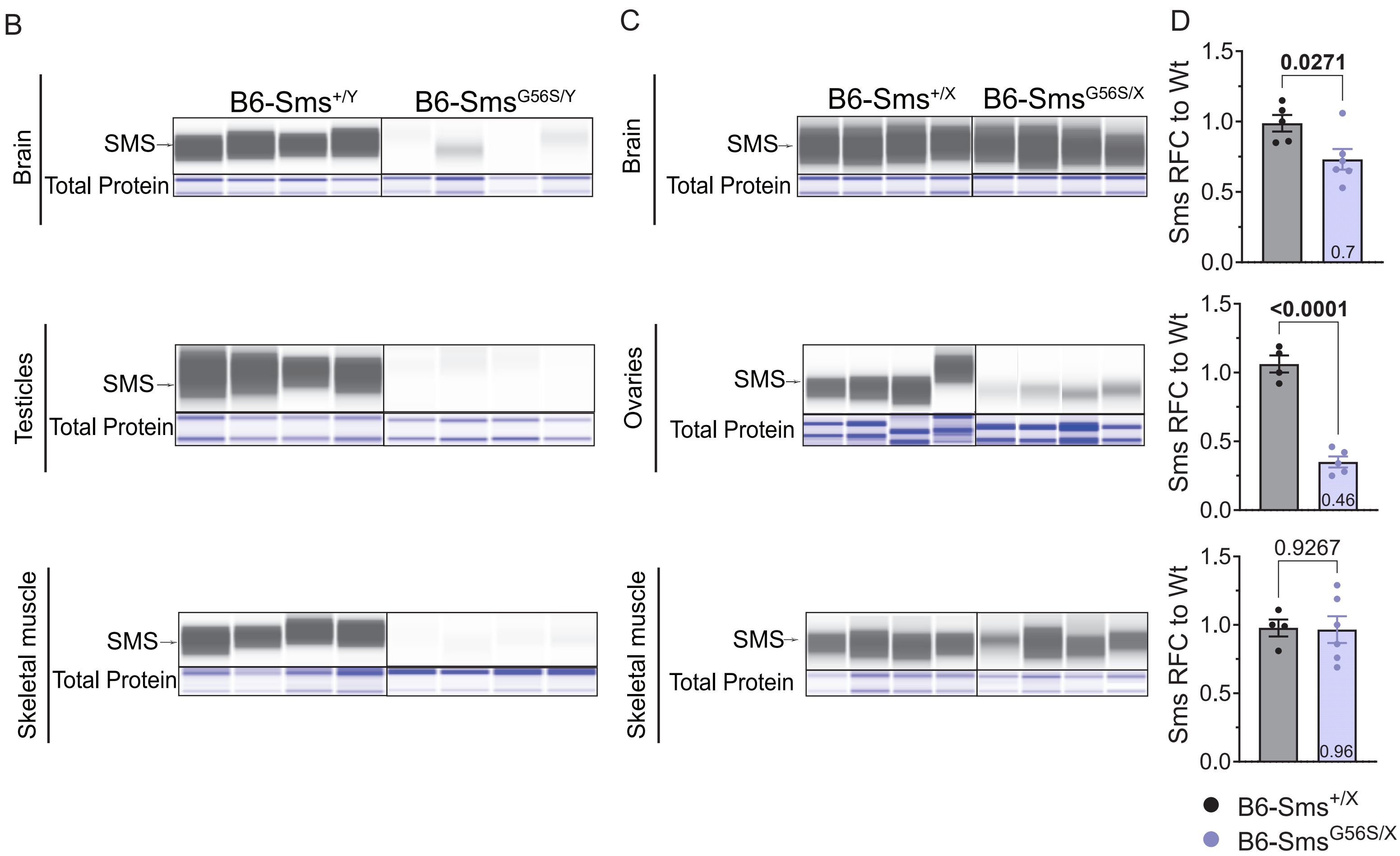

Fig. S1. Homology between human and mouse SMS proteins and effects of the G56S allele on SMS protein expression. (**A**) Sequence alignment of human and mouse spermine synthase proteins indicates 96% identity (366 amino acids total). (**B**) SimpleWes representation of SMS protein (~45 kDa) in tissues (brains, testicles, skeletal muscle) of 8-week-old B6-Sms$^{+/Y}$ (WT) and B6-Sms$^{G56S/Y}$ hemizygous males.(**C**) SimpleWes representation of SMS protein assessed by automated western blot in brain, ovaries and skeletal muscle of heterozygous female carriers: B6-Sms$^{G56S/X}$ (n = 6 for brain and skeletal muscle, n = 5 for ovaries) compared to WT littermates: B6-Sms$^{+/X}$ (n = 5 for brain; n = 4 for ovaries and skeletal muscle) at 8 weeks old. (**D**) Peak area for SMS was normalized to total protein, and fold-change was calculated relative to WT. Bars represent the means ± s.e.m.; dots indicate individual mice. Statistical analysis was using unpaired t-test.

Disease Models & Mechanisms • Supplementary information

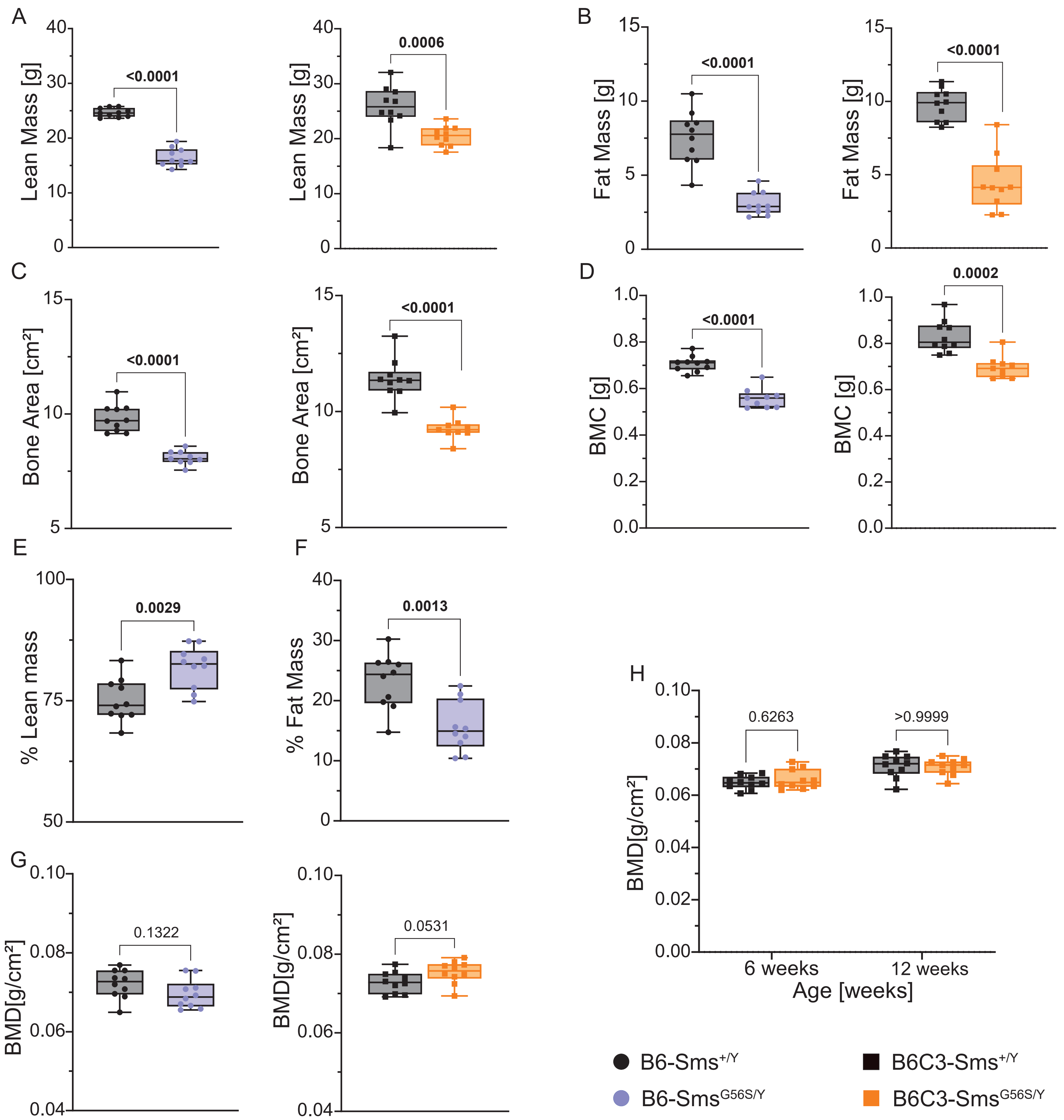

Fig. S2. Alterations in body weight and composition in the B6 and B6C3-Sms^{G56S} mouse models. Dual-energy X-ray absorptiometry (DEXA) was performed on B6-Sms$^{+/Y}$ and B6-Sms$^{G56S/Y}$ as well as the mixed background B6C3-Sms$^{+/Y}$ and B6C3-Sms$^{G56S/Y}$ mice at 20 weeks of age; n = 10 mice/genotype. (**A-D**) Raw measurements of lean mass (A), fat mass (B), bone area (C), and bone mineral content (BMC, D). Columns represent the mean determinations of fat mass and lean mass (in grams), bone area (cm^2), and bone mineral content (g) for each genotype, unadjusted for total body weight, at each time point. (**E-F**) Calculated % lean mass (E) and % fat mass (F) as a portion of total body weight at 20 wks. (**G-H**) Calculated bone mineral density (BMD) in g/cm^2 at 20 weeks in B6-Sms mice (G) and 6, 12, and 20 weeks in B6C3-Sms mice (H).

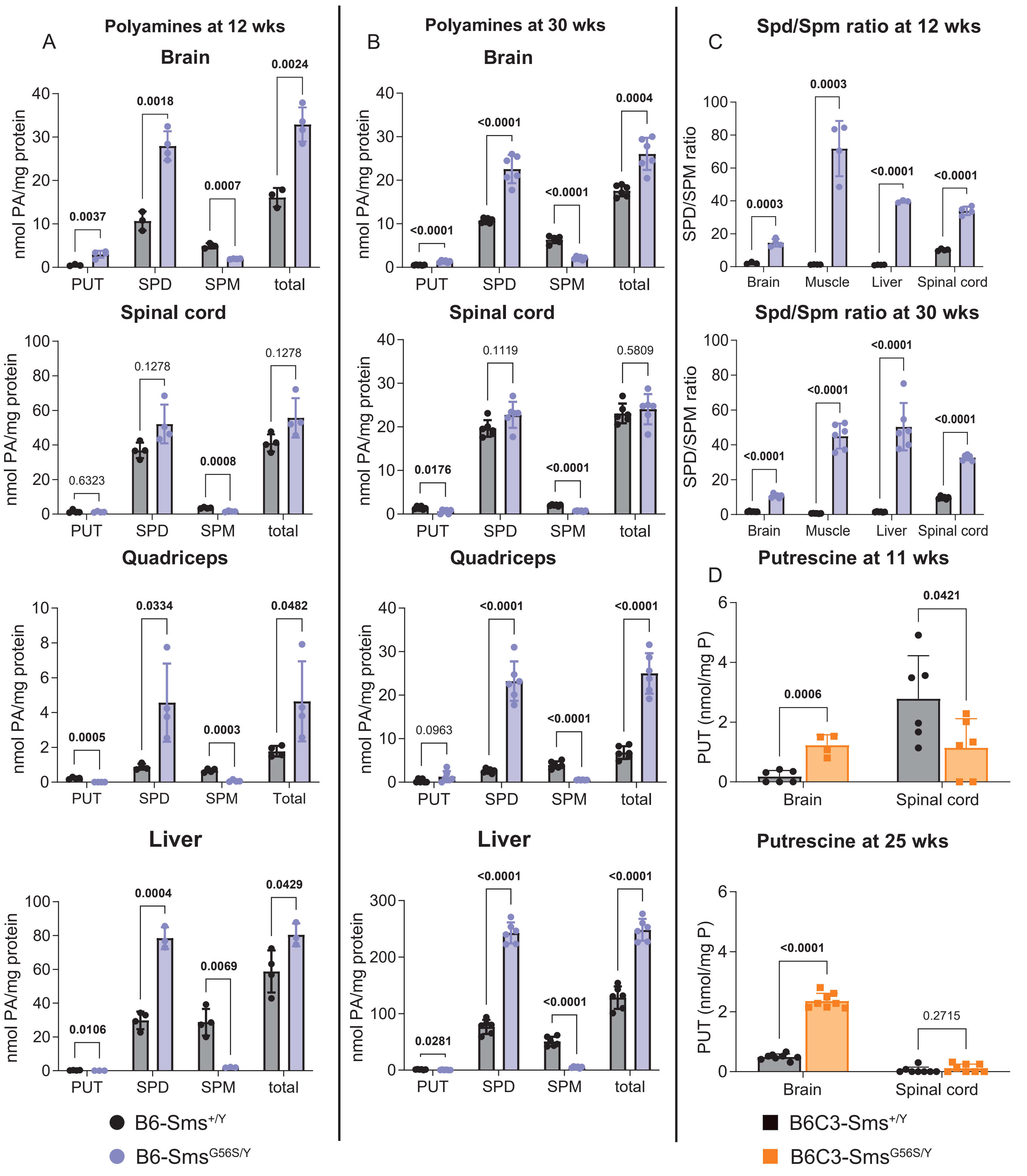

Fig. S3. Intracellular polyamine pool alterations in Sms$^{G56S/Y}$ mouse tissues. Concentrations of putrescine (PUT), spermidine (SPD), spermine (SPM), total polyamines, and the SPD/SPM ratios were determined in WT and SRS (B6-Sms$^{+/Y}$ and B6-Sms$^{G56S/Y}$, respectively) mouse tissues, including brain, spinal cord, quadriceps, and liver. Tissues were collected at 12 (n = 4 per genotype; column A) and 30 weeks of age (n = 6 per genotype; column B). Ordinant units are nmol polyamine (PA) measured with normalization to total cellular protein. Resulting SPD/SPM ratios were calculated for each age group (C). Tissue-specific PUT concentrations in B6C3-Sms mice at 11 (n = 6 in wildtype brain, spinal cord, and SRS spinal cord; n = 4, SRS brain) and 25 weeks (n = 8 per genotype) (D). Column heights indicate the means of each group with s.e.m. Statistical significance was determined individually for each polyamine, total polyamines, and SPD/SPM ratio using multiple, unpaired t-tests. P values smaller than 0.05 are considered statistically significant and are indicated on plots.

Disease Models & Mechanisms • Supplementary information

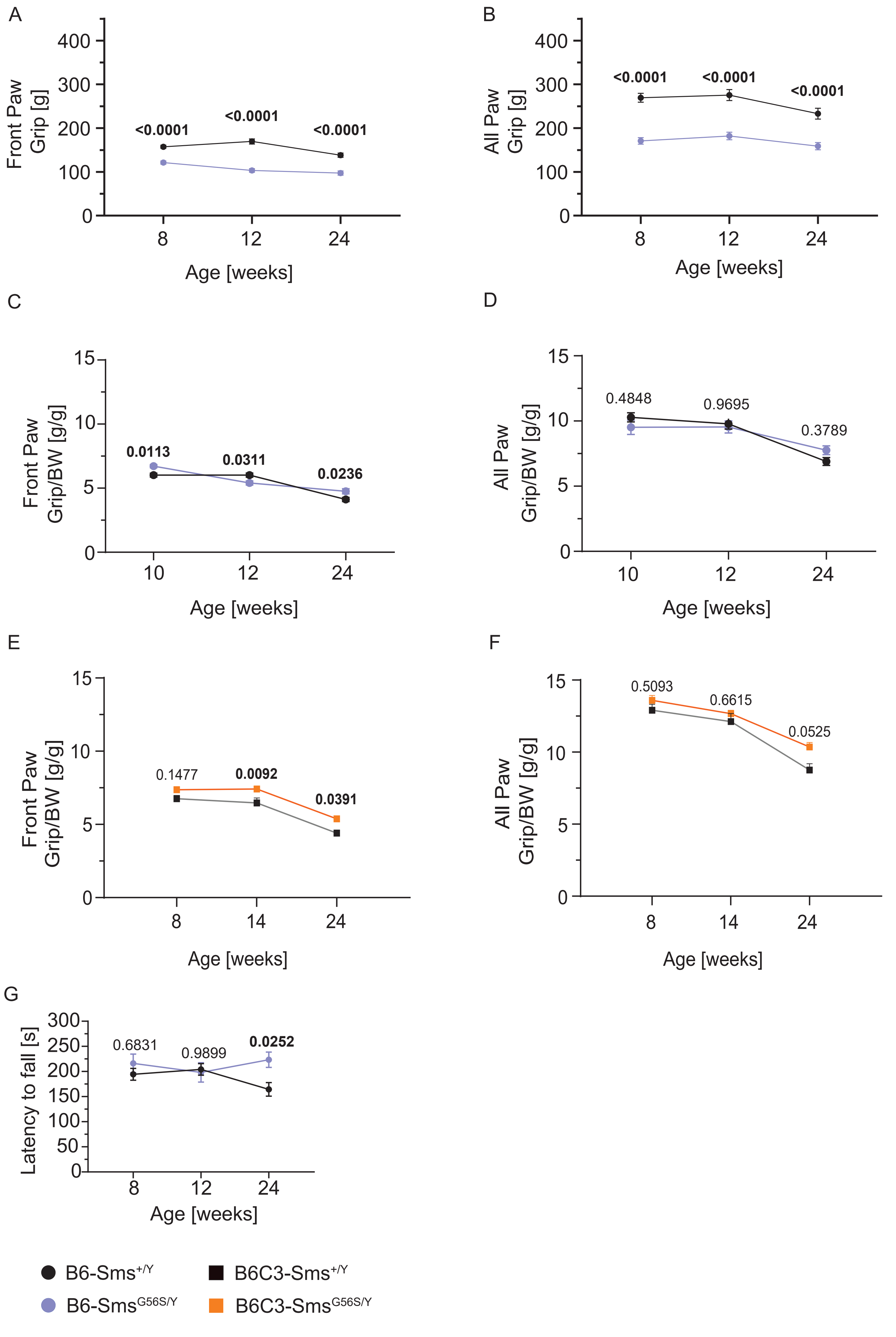
A
Front Paw Grip [g]
<0.0001
<0.0001
<0.0001
Age [weeks]
B
All Paw Grip [g]
<0.0001
<0.0001
<0.0001
Age [weeks]
C
Front Paw Grip/BW [g/g]
0.0113
0.0311
0.0236
Age [weeks]
D
All Paw Grip/BW [g/g]
0.4848
0.9695
0.3789
Age [weeks]
E
Front Paw Grip/BW [g/g]
0.1477
0.0092
0.0391
Age [weeks]
F
All Paw Grip/BW [g/g]
0.5093
0.6615
0.0525
Age [weeks]
G
Latency to fall [s]
0.6831
0.9899
0.0252
Age [weeks]
B6-Sms+/Y
B6C3-Sms+/Y
B6-SmsG56S/Y
B6C3-SmsG56S/Y

Fig. S4. Effects of the Sms^{G56S} allele on neuromuscular function. Neuromuscular function was assessed by measuring forepaw and all-paw grip strength and latency to fall from the rotarod in B6-$Sms^{+/Y}$ and B6-$Sms^{G56S/Y}$ mice at 10, 12, and 24 weeks of age, and in B6C3-$Sms^{+/Y}$ and B6C3-$Sms^{G56S/Y}$ mice at 8, 14, and 24 weeks of age. Grip strength data are shown as raw values for B6 mice (A–B), and as body-weight-adjusted values (B6 in C-D, B6C3 in E-F). Motor coordination was evaluated using the rotarod test in B6-$Sms^{+/Y}$ and B6-$Sms^{G56S/Y}$ mice at 8, 12, and 24 weeks of age (G). Longitudinal rotarod data are presented as mean latency to fall (seconds) ± s.e.m. at each time point. Grip strength data are presented as mean ± s.e.m.; n = 10 mice per genotype for B6 mice and n = 15 mice per genotype for B6C3 mice. Significant P values are indicated in bold on the graphs. Statistical significance was assessed using two-way ANOVA with Šidák's multiple-comparisons correction.

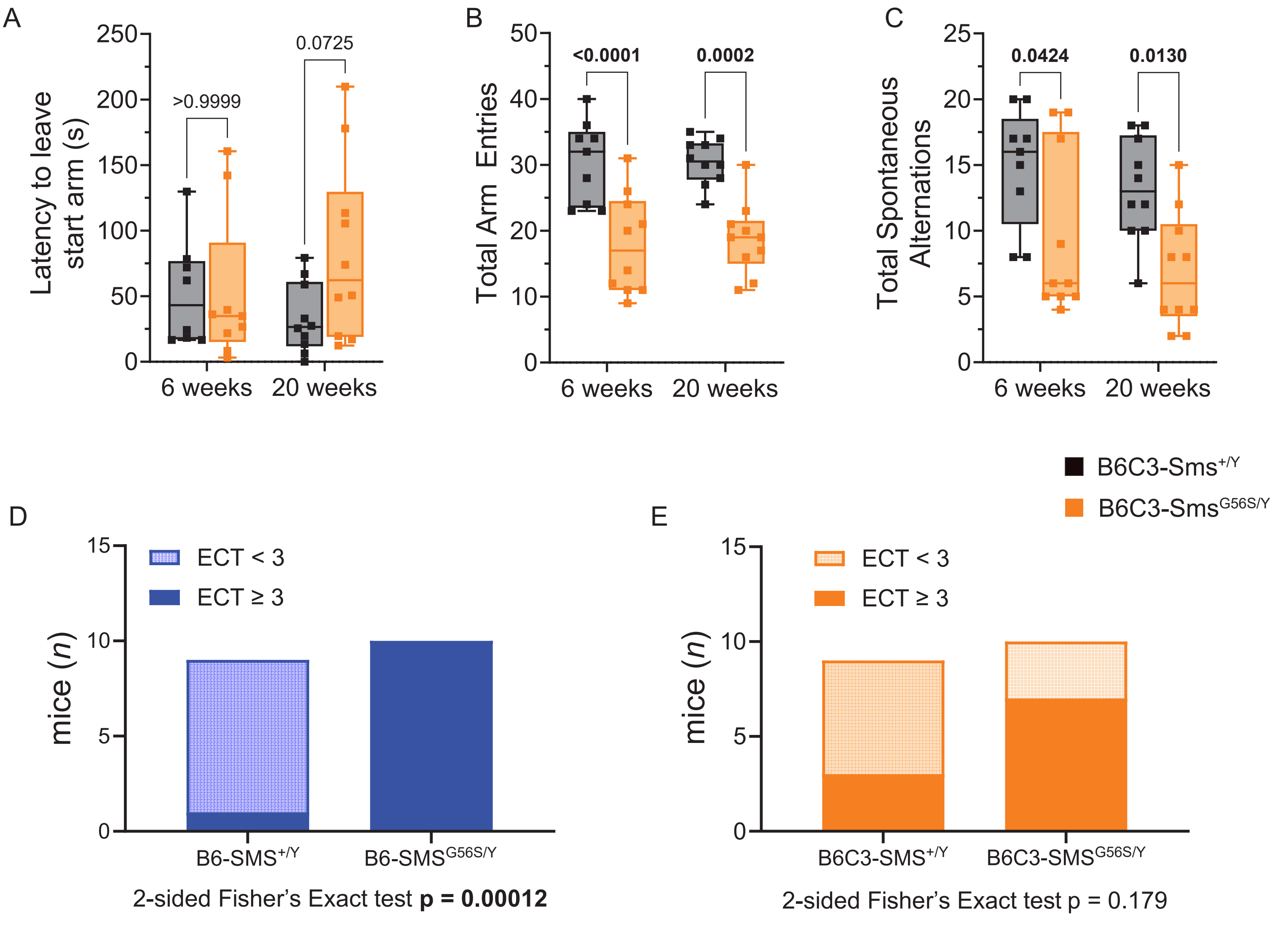

Fig. S5. Analyses of short-term memory and electroconvulsive threshold in SmsG56 mice. (**A-C**) Short-term memory was measured using the Y-maze in B6C3-Sms$^{+/Y}$ and B6C3-Sms$^{G56S/Y}$ mice at 6 and 20 weeks of age. Box & whiskers plots represent latency to leave the start arm (A), total number of arm entries (B), and number of spontaneous alternations (C). (**D-E**) Electroconvulsive threshold (ECT) was performed at 40 weeks of age on wildtype and SmsG56S male mice on both B6 (D) and B6C3 (E) backgrounds; n = 9-10. The proportion of mice exhibiting seizure activity scoring ≥ 3 during the 10-day testing period (up to 11 mA) is indicated as dark blue or orange for the individual strains. Statistical significance was assessed using 2-sided Fisher's exact test, with p-values indicated on graphs.

Disease Models & Mechanisms • Supplementary information

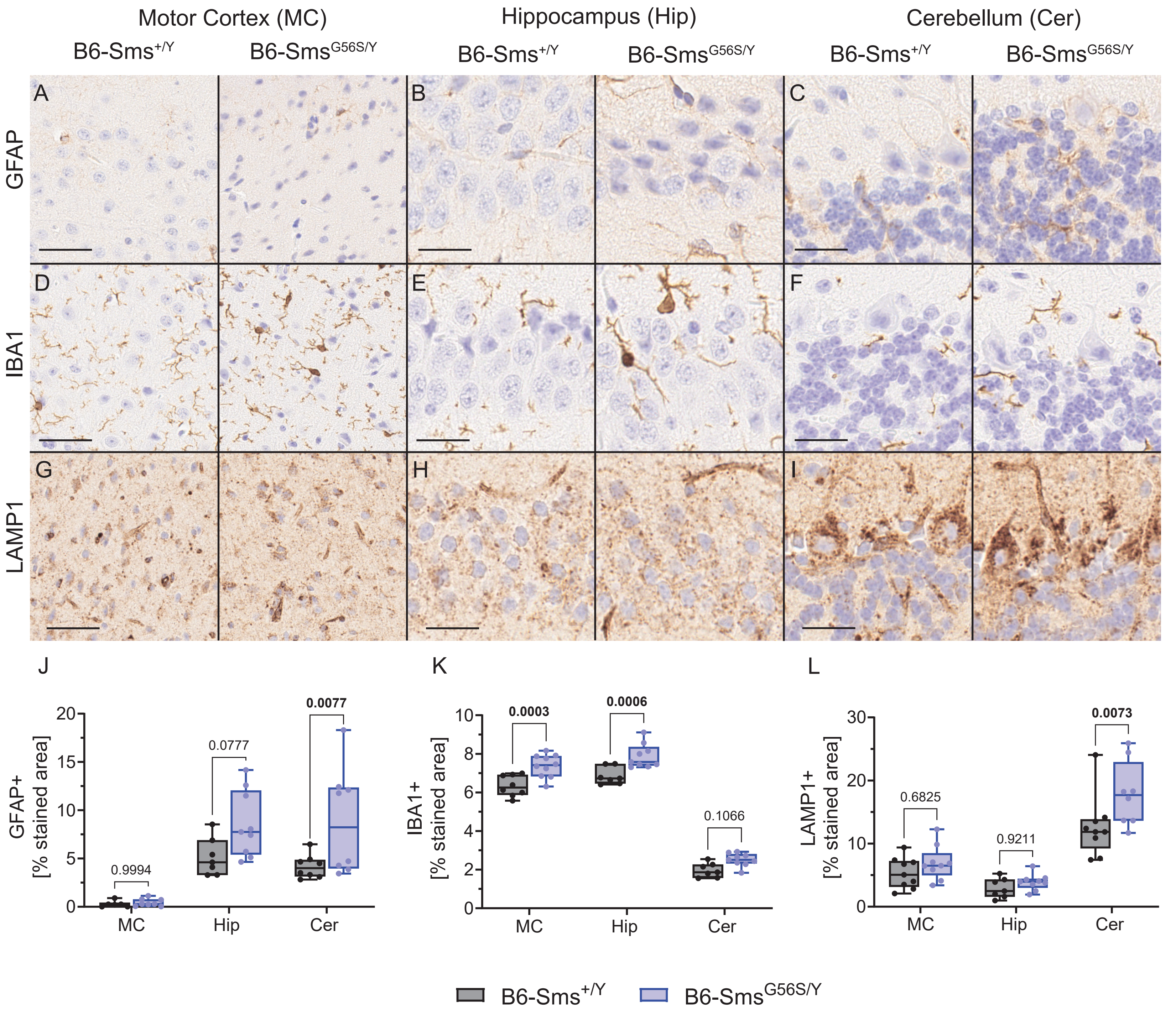

Fig. S6. Effects of the SmsG56S allele on brain immunohistochemistry in B6-Sms mice. Representative GFAP, IBA1 and LAMP1 immunohistochemistry on brain regions representative of motor cortex (A, D, G), hippocampus (CA1) (B, E, H) and cerebellum (C, F, I) at 40 weeks in B6-Sms$^{G56S/Y}$ and B6-Sms$^{+/Y}$ mice. Image magnification: motor cortex at 20x, scale bar at 25 µm, hippocampus and cerebellum at 40x, scale bar at 50 µm. Quantification (J-L) represents the percentage of stained area in motor cortex (A, D, G), hippocampus (B, E, H), and cerebellum (C, F, I) over total area shown. Data are represented as interleaved box and whiskers plots with median, 1st quartile, 3rd quartile, min, and max indicated in individual plots. Multiple comparisons were performed using 2-way ANOVA tests with Šidák correction. Exact P values for comparisons are indicated on plots.
